# A genome-wide survey of sRNAs in the symbiotic nitrogen-fixing alpha-proteobacterium *Sinorhizobium meliloti*

**DOI:** 10.1186/1471-2164-11-245

**Published:** 2010-04-17

**Authors:** Jan-Philip Schlüter, Jan Reinkensmeier, Svenja Daschkey, Elena Evguenieva-Hackenberg, Stefan Janssen, Sebastian Jänicke, Jörg D Becker, Robert Giegerich, Anke Becker

**Affiliations:** 1 Institute of Biology III, Faculty of Biology, University of Freiburg, Freiburg, Germany; 2 Faculty of Technology, Bielefeld University, Bielefeld, Germany; 3 Center for Biotechnology, Bielefeld University, Bielefeld, Germany; 4 Institut für Mikrobiologie und Molekularbiologie, Gießen, Germany; 5 Affymetrix Core Facility, Instituto Gulbenkian de Ciencia, Oeiras, Portugal

## Abstract

**Background:**

Small untranslated RNAs (sRNAs) are widespread regulators of gene expression in bacteria. This study reports on a comprehensive screen for sRNAs in the symbiotic nitrogen-fixing alpha-proteobacterium *Sinorhizobium meliloti *applying deep sequencing of cDNAs and microarray hybridizations.

**Results:**

A total of 1,125 sRNA candidates that were classified as trans-encoded sRNAs (173), cis-encoded antisense sRNAs (117), mRNA leader transcripts (379), and sense sRNAs overlapping coding regions (456) were identified in a size range of 50 to 348 nucleotides. Among these were transcripts corresponding to 82 previously reported sRNA candidates. Enrichment for RNAs with primary 5'-ends prior to sequencing of cDNAs suggested transcriptional start sites corresponding to 466 predicted sRNA regions. The consensus σ^70 ^promoter motif CTTGAC-N_17_-CTATAT was found upstream of 101 sRNA candidates. Expression patterns derived from microarray hybridizations provided further information on conditions of expression of a number of sRNA candidates. Furthermore, GenBank, EMBL, DDBJ, PDB, and Rfam databases were searched for homologs of the sRNA candidates identified in this study. Searching Rfam family models with over 1,000 sRNA candidates, re-discovered only those sequences from *S. meliloti *already known and stored in Rfam, whereas BLAST searches suggested a number of homologs in related alpha-proteobacteria.

**Conclusions:**

The screening data suggests that in *S. meliloti *about 3% of the genes encode trans-encoded sRNAs and about 2% antisense transcripts. Thus, this first comprehensive screen for sRNAs applying deep sequencing in an alpha-proteobacterium shows that sRNAs also occur in high number in this group of bacteria.

## Background

Since the discovery of the first small non-coding RNA (sRNA) in 1981, this class of untranslated transcripts of 50 to 514 nucleotides (nt) in length has become more and more evident in transcriptional and posttranscriptional regulation in prokaryotes [1-3]. In addition to tmRNA, 4.5S RNA, 6S RNA, and RNAseP, which are related to house-keeping gene expression, a number of additional sRNAs was identified. These are broadly classified in two major populations, (i) cis-encoded antisense sRNAs, oriented antisense to their target genes and (ii) trans-encoded sRNAs situated in distinct locations from their targets [4-8]. sRNA-mediated posttranscriptional regulation was characterized in a variety of cell processes, e.g. transposition [9], bacterial virulence [10], quorum sensing [10,11], plasmid replication [12], function of toxin-antitoxin systems [13], and responses to different stress conditions [14].

Cis- and trans-encoded sRNAs each function in a different manner by interacting with short regions of mRNA transcripts via perfect and imperfect sequence complementarity, respectively [7]. The main mechanisms of sRNA-mediated control of gene expression are: repression [15-17] or activation of translation [18], mRNA degradation [19,20] or stabilization [21], and target mimicry [7,22]. sRNAs may act in different ways on different targets, e.g. the *E. coli *RyhB sRNA is a translational activator of *shiA *and a repressor of *sodB *mRNA [23,24].

In addition, 5'-untranslated regions of bacterial mRNA were found that regulate transcription attenuation and translation initiation in response to levels of specific metabolites or intracellular temperature [25,26]. These mRNA regions are called riboswitches. The metabolite effectors are generally able to mediate changes between alternative secondary structures by binding to a metabolite sensing domain of the RNA which prevents translation. In contrast, RNA thermometers are structures sensitive to temperature shifts. Usually, these elements are located in the 5'-UTR (untranslated region) including the ribosomal binding site (RBS). Increasing of the temperature permits destabilization of the secondary structure and releases the RBS for translation [25,27]. For example ROSE-like RNA thermometers (Repression of heat-Shock gene Expression) are widespread in alpha- and gamma-proteobacteria and two putative candidates were found in *Sinorhizobium meliloti *[25].

To date, genome-wide profiling of sRNAs by experimental approaches was undertaken in several Gram-positive and Gram-negative bacteria [28]. However, comprehensive experimental sRNA screening data is not yet available for the group of alpha-proteobacteria, with the exception of a tiling microarray-based transcriptome study in *Caulobacter crescentus *[29]. Our study aimed at a genome-wide discovery of sRNAs in *Sinorhizobium meliloti *that belongs to the *Rhizobiales *of the alpha-proteobacteria. *S. meliloti *exists either in symbiosis with its leguminous host plants (e.g. *Medicago sativa*) or in a free-living lifestyle. The bacteria associate with the plant root and induce the formation of nodules that become colonized by the bacteria via infection threads. Inside the nodule, the bacteria differentiate into bacteroids that are capable of nitrogen-fixation to the benefit of the host plant [30]. The genome of *S. meliloti *is composed of one chromosome (3.65 Mb, 3351 predicted protein-encoding genes) and two megaplasmids, pSymA (1.35 Mb, 1291 predicted protein-encoding genes) and pSymB (1.68 Mb, 1583 predicted protein-encoding genes) [31,32].

In addition to 4.5S RNA, tmRNA, and RNAseP [31,33,34], the cis-encoded antisense sRNAs IncA and SuhB were previously identified in *S. meliloti *and related alpha-proteobacteria [12,35,36]. IncA mediates the posttranscriptional repression of the replication initiation protein-encoding gene *repC*, located in the *repABC *operon. This highly conserved operon is essential for replication, segregation and copy number of many extrachromosomal replicons in alpha-proteobacteria, e.g. the symbiotic megaplasmids in *S. meliloti*, the tumor inducing plasmid in *A. tumefaciens *and the second chromosome in *Brucella *[12]. SuhB was first discovered in *A. tumefaciens *in opposite orientation to the *suhB *gene encoding an inositol-monophosphatase [35]. In *S. meliloti*, four *suhB *paralogs were identified [34].

Three recent studies primarily applied bioinformatics approaches to the identification of sRNA candidates in *S. meliloti*. del Val *et al*. [37] employed a genome wide computational analysis of *S. meliloti *intergenic regions leading to 32 candidates, eight of which were experimentally confirmed. Ulvé *et al*. [38] discovered 14 novel sRNAs combining several computational approaches with microarray as well as Northern and dot blot hybridizations for validation. Computational predictions and microarray hybridization experiments were also combined by Valverde *et al*. [39] to screen the intergenic regions resulting in 14 candidates that were confirmed as novel small non-coding RNAs by Northern blot and/or microarray hybridizations.

In this study, we have performed a comprehensive experimental screening for sRNAs in *S. meliloti *applying deep sequencing technologies as well as oligonucleotide microarray and chip hybridizations. This approach resulted in 1,125 transcription units that are novel candidates for trans-encoded sRNAs, cis-encoded antisense sRNAs, sense sRNAs or mRNA leader transcripts suggesting that in *S. meliloti *about 3% of the genes encode trans-encoded sRNAs and about 2% antisense transcripts. Expression patterns provided further information on conditions of expression of a number of sRNA candidates. Sequence conservation analyses suggest strong similarities of a subset of *S. meliloti *sRNAs to regions in related alpha-proteobacteria.

## Results

### sRNAs identified by deep sequencing

#### Data generation by 454 and Illumina/Solexa sequencing of cDNAs

A considerable proportion of sRNAs is probably only transcribed at high levels under specific conditions. To increase the probability of discovery of these sRNAs by our screening approach, small RNA fractions from a number of conditions were pooled for deep sequencing. These included exponential and stationary growth phases as well as shifts to low or high temperature, to low or high pH, to high salt concentration, and addition of H_2_O_2 _to cause oxidative stress (see "Methods" for details).

**Figure 1:**
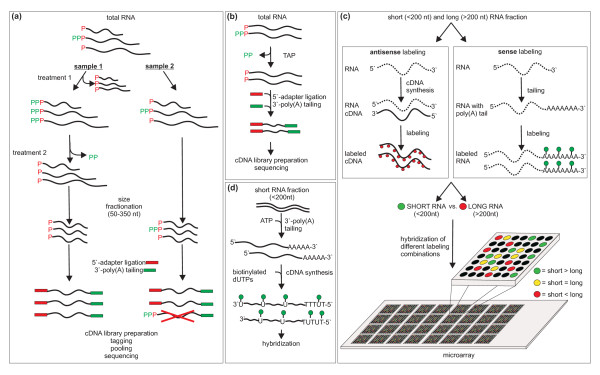
**Experimental procedures for non-coding sRNA identification**. **(a) **Sample preparation for deep sequencing with GS FLX: Sample 1 is enriched for primary transcripts. Treatment 1: Terminator Phosphate Dependent Exonuclease (TPE) was used to eliminate processed transcripts. Treatment 2: Tobacco Acid Pyrophosphatase (TAP) was used to eliminate pyrophosphates from primary transcripts. Sample 2 is enriched for processed transcripts. **(b) **Sample preparation for deep sequencing with Genome Analyzer II. **(c) **Sample labeling and hybridization for microarray-based screening. **(d) **Sample preparation for Affymetrix Symbiosis Chip-based screening.

**Figure 2:**
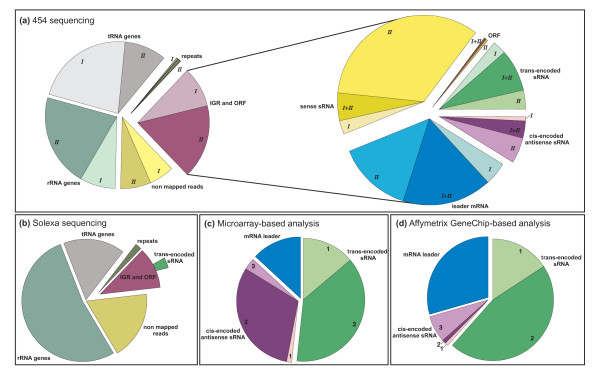
**Relative proportion of sRNA candidates in different classes**. **(a) **454 sequencing: distribution of reads mapped to the *S. meliloti *1021 genome and distribution of the analyzed contigs according to the general classification (Figure 3). Left circle diagram: light colored (I) and colored (II), number of reads derived from sample 1 and 2. Reads in sample 1 and 2: non-mapped, 48,159 and 57,964; rRNA genes, 67,891 and 176,848; tRNA genes, 188,121 and 79,789; repeats, 3,029 and 6,206; IGRs or ORFs, 77,326 and 140,702. Right circle diagram: light colored (I), colored (II) and dark colored (I+II) represent the number of RNA candidates derived from sample 1, sample 2, and both samples, respectively: trans-encoded sRNAs, 28, 38, 85; cis-encoded antisense sRNAs, 9, 52, 35; mRNA leader transcripts, 46, 151, 181; sense sRNAs 28, 363, 56; ORFs 0, 4, 4. **(b) **Illumina/Solexa sequencing: Distribution of reads mapped to the *S. meliloti *1021 genome. Reads: non-mapped, 1,179,722; rRNA genes, 3,405,289; tRNA genes, 1,058,534; repeats, 111,355; IGR and ORFs, 711,851. Dark green segment: contigs for 44 putative trans-encoded sRNAs. **(c) **Microarray-based analysis and **(d) **Affymetrix Symbiosis Chip-based analysis: distribution of sRNA candidates. Segment numbers represent subtypes. Microarray data: type 1 and 2 trans-encoded sRNAs, 264 and 721 candidates; type 1, 2 and 3 cis-encoded antisense sRNAs, 25, 587 and 59; mRNA leader transcripts, 250. Affymetrix Symbiosis Chip data: type 1 and 2 trans-encoded sRNAs, 60 and 174; type 1, 2 and 3 cis-encoded antisense sRNAs, 3, 4 and 27; mRNA leader, 112.

Two small RNA samples which were enriched for primary (sample 1) and processed transcripts (sample 2) (Figure 1a) were fractionated to a size range of 50 to 350 nt and subjected to 454 GS FLX Titanium sequencing. A total of 384,526 and 461,509 reads were generated from sample 1 and 2, respectively. Following the mapping to the *S. meliloti *1021 reference genome, the reads were matched to their original strand taking advantage of the sequence of the 5'-RNA adapter used in sample preparation. Approximately 70% of all reads were mapped to rRNA- or tRNA-encoding genes, or to repeat regions (Figure 2a). Excluding the reads that did not map to the genome, the remaining 218,028 reads were assigned to either intergenic regions (IGR), open reading frames (ORF), or regions overlapping both (Figure 2a).

In addition to 454 sequencing of the pool of small RNA fractions obtained from the different conditions, the pool of total RNA was subjected to Illumina/Solexa sequencing (Figure 1b). Approximately 5.3 million reads (out of 6.5 million) were mapped to the reference genome (Figure 2b). After removal of the reads that mapped to repeat regions, including the rRNAs and tRNAs, the remaining 711,851 reads were uniquely mapped either to ORFs or IGRs.

#### Transcript definition

Low abundant reads that may have originated from transcriptional background and mRNA degradation were filtered-out by the following selection criteria employed to determine the contigs for further analyses. We defined a contig by a seed region of length L, covered by at least C reads. The seed region was extended on either side as long as read coverage was at least c. For 454 reads, we used: L = 50-350, C = 10, c = 5. 1,111 contigs were identified, with the majority (960) associated to coding regions and the remaining 151 contigs mapping to IGRs (Figure 2a). For the shorter Illumina/Solexa reads we used: L = 50, C = 5, c= 2. 1,012 contigs were identified, with the majority (968) located inside coding regions and the remaining 44 contigs mapping to IGRs (Figure 2b). These contigs were taken as the observed units of transcription in further analyses.

#### Transcript classification

**Figure 3:**
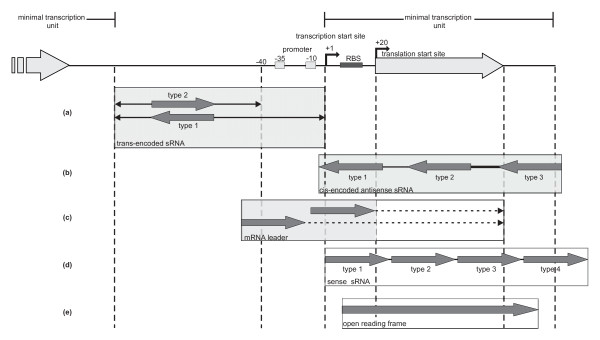
**Classification of 454 contigs**. Contig classification is based on a model of a minimal transcription unit. RBS, ribosomal binding site. Five classes were defined: (**a**) trans-encoded sRNAs are located at least 60 nt upstream and 20 nt downstream from the translation start and stop codons, respectively. Type 1 is located antisense to both adjacent genes, type 2 sRNAs are flanked by at least one adjacent gene in the same orientation. (**b**) Cis-encoded antisense sRNA in the opposite direction of the minimal transcription unit grouped into type 1-3 depending on the relative location to the associated gene. Type 1, 2 and 3 are located antisense to the 5'-UTR, to the coding region and to the 3'-UTR, respectively. (**c**) mRNA leader sequences either overlap the 40 nucleotides upstream of the minimal transcription unit or starting between position -40 and +1. The 3'-end of each contig is located inside the open reading frame (dashed line). (**d**) A sense sRNA is located in the same direction as the minimal transcription unit and assigned to one of four subclasses: type 1, 2, 3 and 4 overlaps the 5'-UTR, is located inside the ORF, overlaps the 3'-UTR, and starts inside the 3'-UTR, respectively. (**e**) Open reading frame: A contig that overlaps the whole ORF. The boxes highlighted in grey indicate classes used for classification of candidates derived from the microarray- and Affymetrix Symbiosis Chip-based screenings.

With respect to their positions relative to the neighboring or overlapping ORFs, contigs were grouped into five classes: (i) trans-encoded sRNAs, (ii) cis-encoded antisense sRNAs, (iii) mRNA leader sequences, (iv) sense sRNAs overlapping with ORFs, and (v) transcripts completely covering an ORF (Figure 3, Table 1) [Additional file 1]. We identified eight such ORFs that were previously identified as genes encoding small proteins (Table 1) [Additional file 1] [31,32,40]. Putative sRNA genes were included in the GenDB *S. meliloti *genome project [32]http://www.rhizogate.de.

**Table 1:** Replicon localization and classification of sRNA candidates

		Deep sequencing			Microarray experiments		
**RNA type**	**sub type**	**chromosome**	**pSymA**	**pSymB**	**chromosome**	**pSymA**	**pSymB**

**trans-encoded sRNA**	1	9	3	4	90	113	61
	2	90	19	26	265	281	175
	-*	11	8	3			
	all	**173**			**985**		

**cis-encoded antisense sRNA**	1	8	3	6	8	9	8
	2	12	4	9	114	230	243
	3	30	4	16	21	20	18
	1/3	1	1	2	0	0	0
	all	**96**			**671**		

**mRNA leader**		298	31	49	90	65	95
	all	**378**			**250**		

**sense sRNA**	1	17	5	6			
	2	221	27	37			
	3	89	5	14			
	4	9	0	3			
	1/3	7	0	2			
	1/4	2	0	0			
	2/4	1	1	1			
	all	**447**					

**open reading frame**		8	0	0			
	all	**8**					

RNA type and subtype: Class of transcripts according to the classification in Figure 3. Replicon: C, chromosome; A, pSymA; B, pSymB. Number: number of identified sRNAs per class, subclass and replicon. *Candidates derived from Illumina/Solexa sequencing that did not allow for identifying the DNA strand. Therefore, subclassification is missing for these candidates.

Candidates for mRNA leader transcripts are defined as short RNAs that probably represent a stable derivative of the 5'-part of the mRNA of a protein-encoding gene. This may be a processed form or a prematurely terminated mRNA due to attenuation or riboswitch activity. Since the transcription start sites of the majority of the protein-encoding genes are unknown, it cannot be excluded that in some cases a putative mRNA leader rather represents an sRNA transcribed from a promoter different from that of the mRNA. Sense sRNAs represent short stable transcripts whose sequences are located within mRNA sequences. Most of these sense sRNAs were probably processed from mRNAs.

Cis-encoded antisense sRNAs were further sub-classified into three groups depending on their relative positions in antisense to the 5'- (type 1) and 3'-UTR (type 3), or inside an ORF (type 2) (Figure 3). Sense sRNAs were further divided into candidates situated inside another ORF (type 2), transcripts overlapping the 3'-UTR and the ORF (type 3), and regions overlapping only the 3'-UTR (type 4). Sense transcripts overlapping the corresponding ORF, but with a 5'-UTR that was considered to be too short for translation initiation, were classified as type 1 sense sRNAs and not as putative mRNA leader transcripts (Figure 3). Trans-encoded sRNAs were sub-classified as type 1 if their orientation is antisense to both neighboring ORFs, and else as type 2 (Figure 3).

Since the Illumina/Solexa sequencing did not allow for identification of the transcribed strand, only trans-encoded sRNAs from this approach were considered for further analyses (Table 1) [Additional file 1]. Taken together, both deep sequencing approaches revealed 173 putative trans-encoded sRNAs. From these 22 were exclusively derived from the Illumina/Solexa sequencing data. sRNA candidates associated to transposable element that often occur in multiple copies are not included in this number and are considered separately for the following analyses.

#### Transcription start sites and 3'-end information retrieved from deep sequencing data

Information on 5'- and 3'-ends of transcripts in sample 1 enriched for primary transcripts and sample 2 enriched for processed transcripts were retrieved from the 454 sequencing data (Table 2) [Additional file 1]. This analysis indicated the positions of transcription start sites and 3'-ends of a number of sRNAs. Two-thirds of the trans-encoded sRNAs displayed one or two distinct 5'-ends. The remaining transcripts possessed more than two or highly variable 5'-end positions which did not allow for identifying the transcription start sites. The proportion of transcripts with one or two distinct 5'-ends was even higher in the class of cis-encoded antisense sRNAs. Only 23% of sRNAs from this class displayed multiple or highly variable 5'-ends. A similar distribution of 5'-ends was observed in the class of mRNA leader transcripts. In the class of sense sRNAs, the proportion of transcripts with one or two defined 5'-ends was 72%.

**Table 2:** 5'- and 3'-end properties of the sRNA candidates

ends		trans-encoded sRNA (151)				cis-encoded antisense sRNA (96)				mRNA leader (378)				sense sRNA (447)			
**5'**	**3'**	**S1**	**S2**	**S1&2**	**total**	**S1**	**S2**	**S1&2**	**total**	**S1**	**S2**	**S1&2**	**total**	**S1**	**S2**	**S1&2**	**total**
**0**	**0**	0	5	0	**5**	0	3	0	**3**	1	10	0	**11**	0	62	0	**62**
	**1**	0	3	0	**3**	0	2	0	**2**	0	3	0	**3**	0	16	0	**16**
	**2**	0	0	0	**0**	0	0	0	**0**	0	0	0	**0**	1	1	0	**2**
	**m**	0	0	0	**0**	0	0	0	**0**	0	0	0	**0**	0	0	0	**0**

**1**	**0**	13	9	6	**28**	7	16	3	**26**	18	69	25	**112**	18	103	12	**133**
	**1**	5	6	9	**20**	2	12	2	**16**	8	12	23	**43**	5	83	10	**98**
	**2**	1	2	3	**6**	0	4	3	**7**	3	5	8	**16**	1	14	3	**18**
	**m**	0	0	12	**12**	0	1	5	**6**	1	2	13	**16**	0	4	1	**5**

**2**	**0**	2	2	6	**10**	0	3	3	**6**	10	17	11	**38**	2	16	1	**19**
	**1**	3	5	2	**10**	0	4	2	**6**	2	13	21	**36**	0	20	5	**25**
	**2**	0	1	3	**4**	0	1	2	**3**	1	4	6	**11**	1	7	4	**12**
	**m**	1	1	12	**14**	0	1	3	**4**	0	1	16	**17**	0	6	4	**10**

**m**	**0**	1	0	2	**3**	0	0	1	**1**	1	2	7	**10**	0	0	1	**1**
	**1**	2	1	2	**5**	0	1	1	**2**	1	8	13	**22**	0	7	5	**12**
	**2**	0	3	6	**9**	0	1	2	**3**	0	5	13	**18**	0	10	2	**12**
	**m**	0	0	22	**22**	0	3	8	**11**	0	0	25	**25**	0	14	8	**22**

Ends: 5' and 3'; number of defined 5'/3'-ends per transcript; S1, S2, S1&2: number of transcripts in sample 1, 2 and both samples, which match the defined 5'/3'-end criteria; total: number of transcripts in all samples which match the defined 5'/3'-end criteria.

Primary 5'-ends were identified for 74%, 46%, and 60% of the trans-encoded sRNAs, antisense sRNAs, and mRNA leader transcripts, respectively. In contrast, only 18% of the transcripts classified as sense sRNAs displayed a primary 5'-end indicating that the majority of these transcripts was processed from mRNAs of the associated protein-encoding gene.

**Figure 4:**
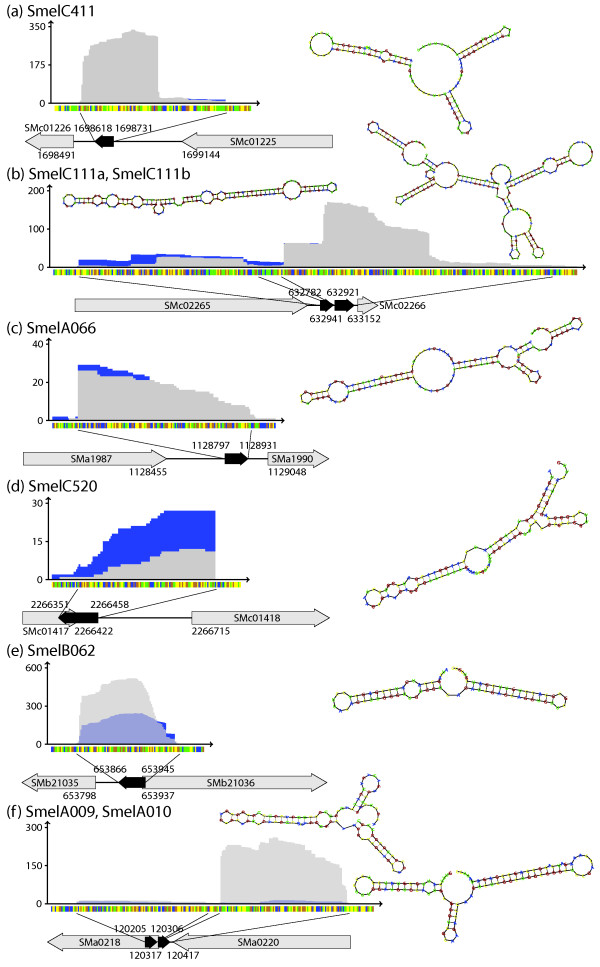
**Examples of sequence profiles and secondary structures of full length trans-encoded sRNAs with common 5'- and 3'-end features**. Sequence coverage profile: blue and light grey color denote transcript coverages derived from sample 1 and 2, respectively. Dark grey colored areas represent an overlap of coverages from both samples. y- and x-axis represent coverage and sequence, respectively. Sequence code: blue, A; yellow, C; orange, G; green, U. Grey arrows represent genes flanking or overlapping sRNA genes. Black arrows represent the sRNAs. **(a) **Trans-encoded sRNA SmelC411, two distinct 5'-ends and one distinct 3'-end; **(b)**trans-encoded sRNA SmelC111a and cis-encoded mRNA leader SmelB111b; three and two distinct 5'-ends, as well as one distinct and a variable 3'-end, respectively; **(c) **trans-encoded sRNA SmelA066, one distinct 5'- and a variable 3'-end; (**d**) type 3 cis-encoded antisense sRNA SmelC520, one distinct 5'-end and a variable 3'-end; (**e**) type 1 cis-encoded antisense sRNA SmelB062, two distinct 5'- and a variable 3'-end; (**f**) type 2 and type 1/3 cis-encoded antisense sRNAs: SmelA009, one distinct 5'-end and a variable 3'-end; SmelA010, several 5'- and 3'-ends.

Comparison of the primary transcript-enriched sample 1 and the processed transcript-enriched sample 2 revealed several different situations with respect to the derived transcript ends [Additional file 1]. Candidates possessing identical 5'-ends were identified in both samples (examples in Figure 4c and 4d). Alternative 5'-ends that may have been caused by posttranscriptional processing of the transcripts or activities of alternative promoters were observed for 403 sRNA candidates (example in Figure 4a, b, and 4e). We also found a number of sRNAs with 5'-ends varying by only one or two nucleotides (example in Figure 4f).

Upstream the transcription start sites of 101 sRNA candidates the σ^70 ^consensus promoter motif CTTGAC-N_17_-CTATAT [41] was predicted [Additional file 2], further confirming the identified primary 5'-ends of these sRNAs. Two promoters of this type were predicted upstream of SmelB154. Only 27 sense sRNA candidates were preceded by this consensus promoter motif, further supporting the assumption that most of these transcripts were processed from mRNAs.

In all classes, about 60% of sRNAs exhibited multiple or highly variable 3'-ends (examples in Figure 4b, c, d, and 4f). The remaining 40% comprised 319 and 121 sRNAs with one or two defined 3'-ends, respectively (examples in Figure 4a, b, and 4e).

In some cases more complex situations were observed. An example is the 371 nt region comprising SmelC111a and SmelC111b (Figure 4b) located in the intergenic region between SMc02265 and SMc02266. This region displayed three putative transcription start sites detected in sample 1 and two 5'-ends that were only found in the processed transcript-enriched sample 2. Furthermore, several 3'-ends were found in this region. This results in separate sRNA transcripts differing in length. Thus, SmelC111a and SmelC111b probably occur as separate sRNAs and were classified as trans-encoded sRNA and leader transcript, respectively.

In 144 cases we found clusters of at least two sRNA candidates separated by less than 200 nt. Among these were nine clusters composed of two and two clusters comprising three trans-encoded sRNAs. Only one protein-encoding gene (SMa0218 encoding a periplasmic solute-binding protein) [31,32,40] was found to be associated with two antisense sRNAs (SmelA009 and SmelA010) (Figure 4f), while approximately 18% of the sense sRNA-associated ORFs include at least two candidates. Interestingly, multiple sRNA candidates were found associated with six of seven genes of the rhizobactin operon.

#### Characteristics of sRNA classes

**Figure 5:**
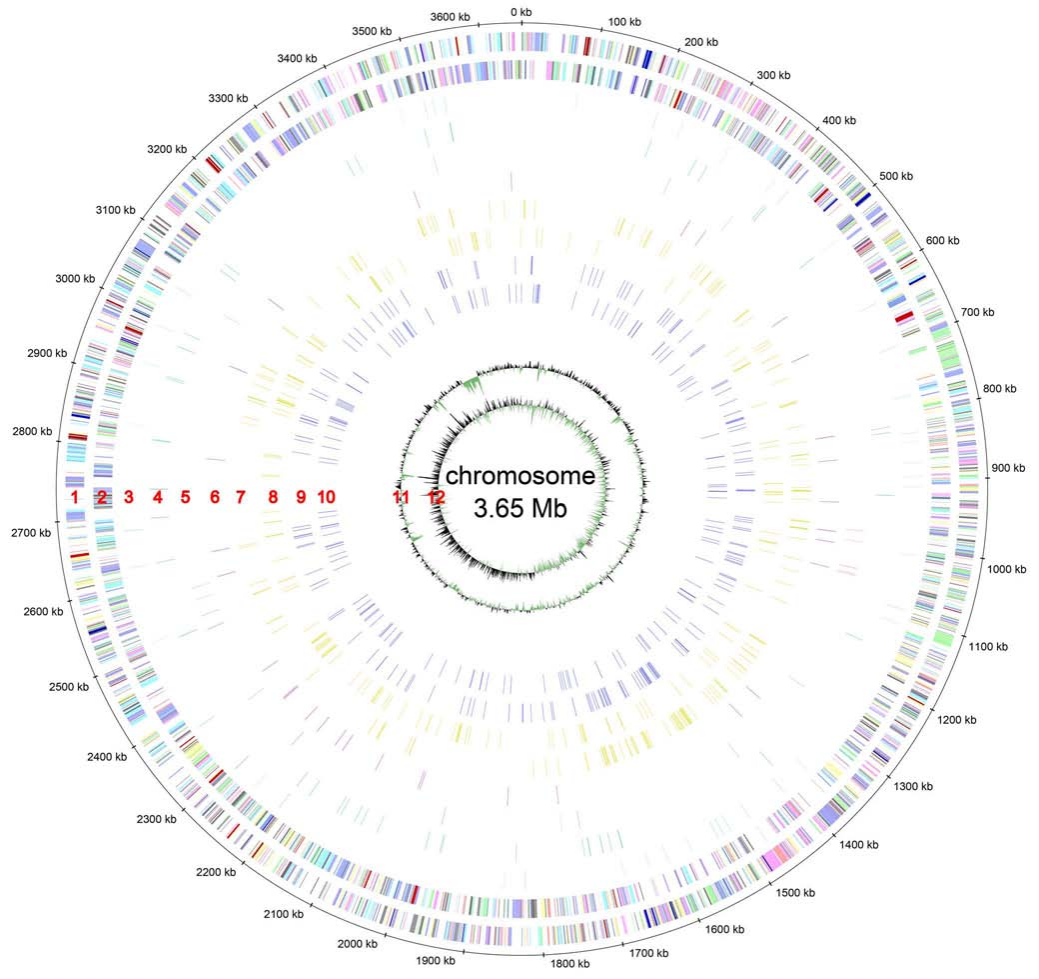
**Genome distribution of sRNA candidates on the chromosome**. sRNA candidates are blotted at their genome position. The outer to inner circles show: 1 and 2, protein-encoding genes on the plus and minus strand, respectively; 3 and 4, trans-encoded sRNAs on the plus and minus strand, respectively; 5 and 6, cis-encoded antisense sRNAs on the plus and minus strand, respectively; 7 and 8, sense sRNAs on the plus and minus strand, respectively; 9 and 10, leader mRNA sequences on the plus and minus strand, respectively; 11 and 12, GC plot and GC skew, respectively.

**Figure 6:**
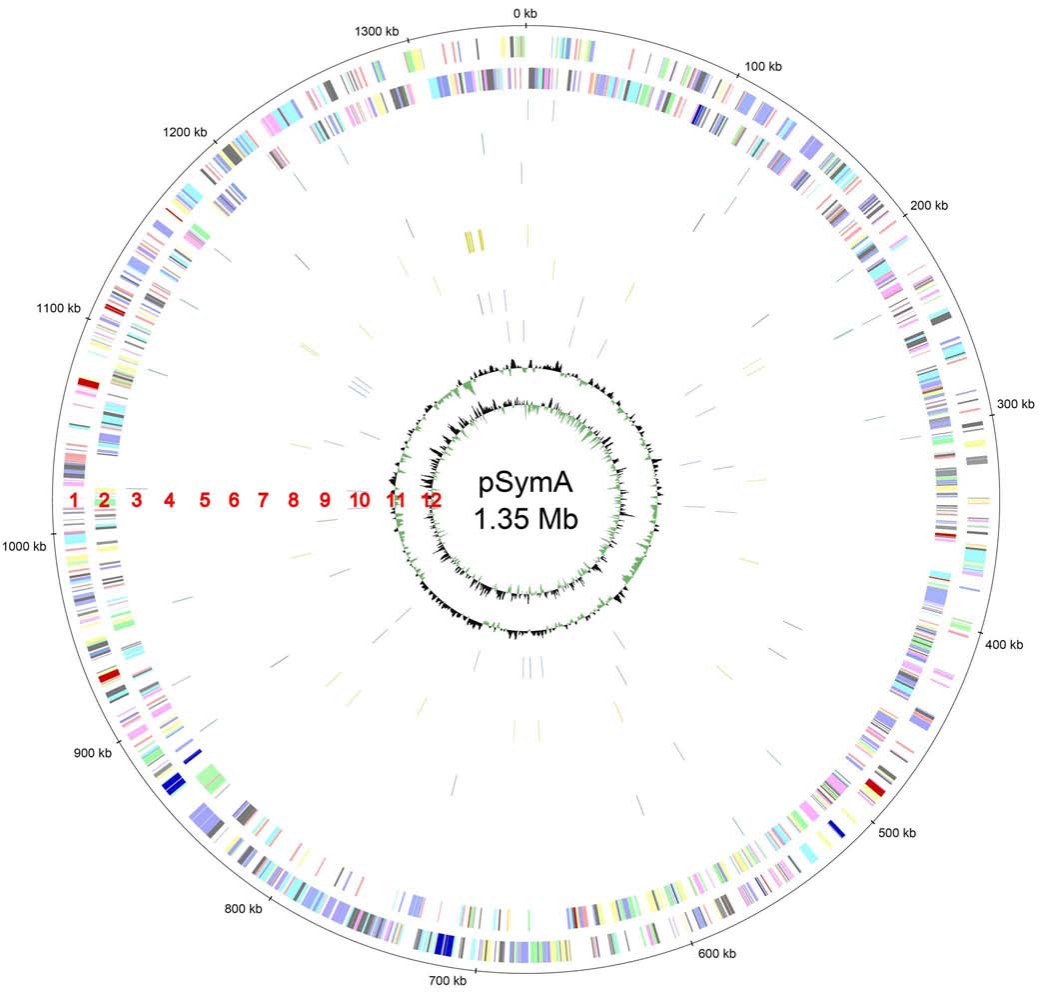
**Genome distribution of sRNA candidates on pSymA**. sRNA candidates are blotted at their genome position. Outer to inner circles: see legend to Figure 5.

**Figure 7:**
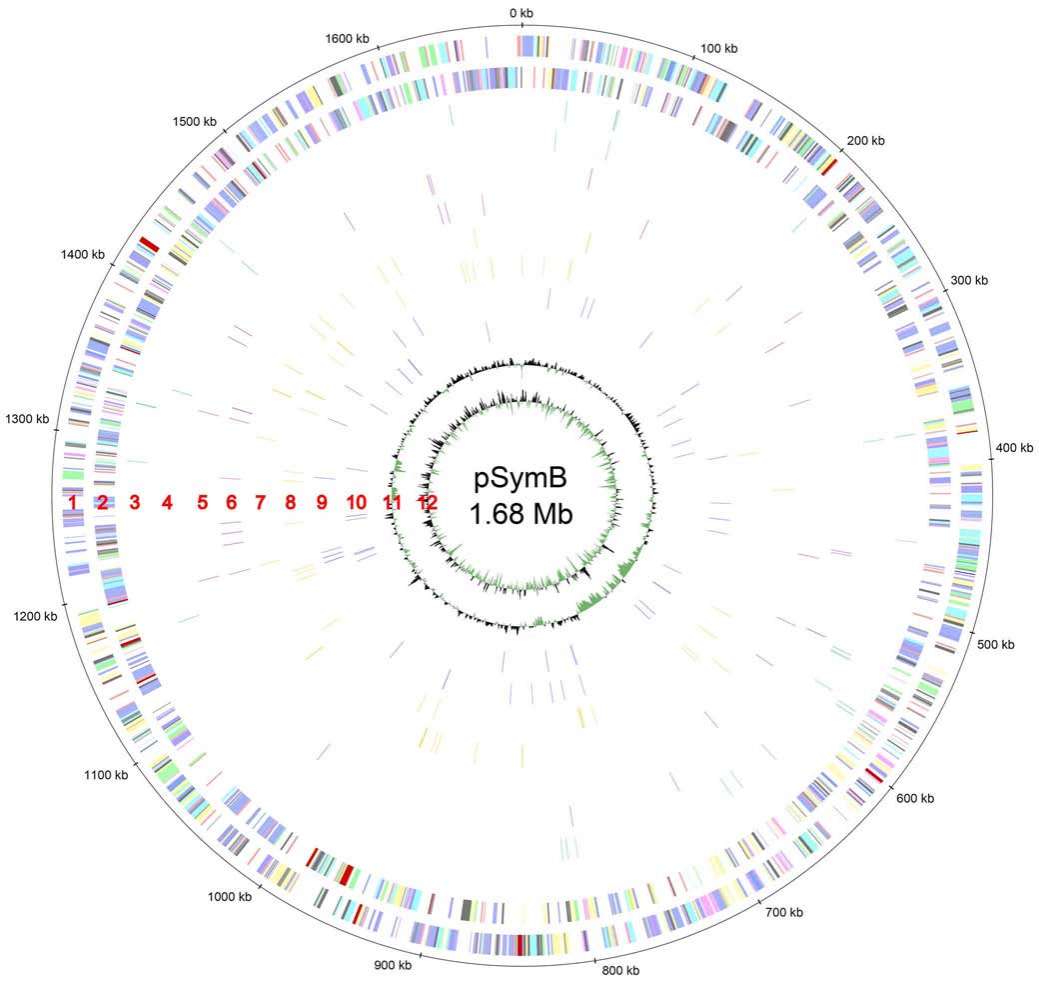
**Genome distribution of sRNA candidates on pSymB**. sRNA candidates are blotted at their genome position. Outer to inner circles: see legend to Figure 5.

The deep sequencing approaches revealed 173 trans-encoded sRNAs, 96 cis-encoded antisense sRNAs, 378 mRNA leader transcripts, and 447 sense sRNA candidates. Distribution of sRNA candidates on the three replicons shows a prevalence of trans-encoded sRNAs, mRNA leader sequences and sense sRNAs on the chromosome (63%, 79%, and 77%, respectively). Between 8% and 22% more sRNAs than expected from an equal distribution of sRNAs (55%) were found on this replicon. In contrast, 9% more cis-encoded antisense RNAs than expected from an equal distribution (25%) were identified on megaplasmid pSymB. sRNA candidate regions were almost evenly distributed over the whole genome (Figures 5, 6, and 7). Thus, clustering was only observed at the gene or intergenic region level.

**Figure 8:**
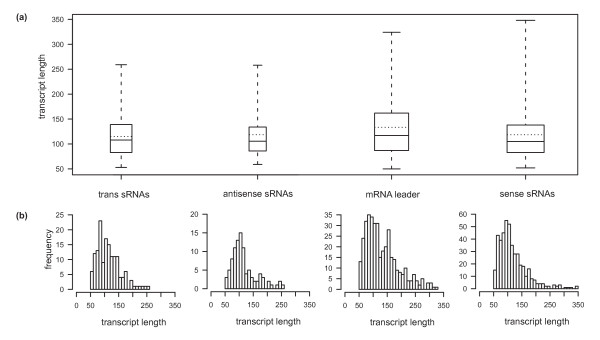
**sRNA length distribution**. (**a**) The box and whisker plot diagram represents the minimum and maximum size, the median as well as the average sizes of the four defined sRNA classes. The sizes of the middle 50% of each candidate population are represented by the lower and upper quartile, respectively. (**b**) The histograms represent the complete length distribution of each individual class.

Figure 8 shows the size distribution of the sRNA candidates deduced from the 454 sequencing data. The trans-encoded sRNAs display an average size of 114 nt with a minimum of 53 nt and a maximum of 259 nt. Box whisker analyses revealed that half of the sRNAs range from 83 to 139 nt in length. Cis-encoded antisense sRNAs display an average length of 117 nt (size range from 59 to 258 nt). Similar to the trans-encoded sRNAs, 50% of these sRNAs vary from 87 to 134 nt in length. The mRNA leader transcripts and sense sRNAs display an average size of 132 nt (size range from 50 to 324 nt) and 118 nt (size range from 52 to 348 nt), respectively. Half of the sense RNAs ranged from 83 to 138 nt in length. In contrast to all other classes, 50% of the sizes of the mRNA leader transcripts range from 87 to 162 nt. sRNAs larger than 350 nt could not be identified in our study due to RNA size fractionation prior to cDNA synthesis.

About half of all antisense sRNA candidates overlap with the 3'-end of the coding region and the 3'-UTR (type 3). 18% overlap with the 5'-UTR (type 1) and 26% are located on the opposite strand inside the ORF (type 2) (Table 1). The majority of sense sRNA candidates are located within the coding region of the associated gene (type 2) (64%) or overlap the 3'-UTR and the ORF (type 3) (24%).

#### Transcription units in repeats and transposable elements

**Figure 9:**
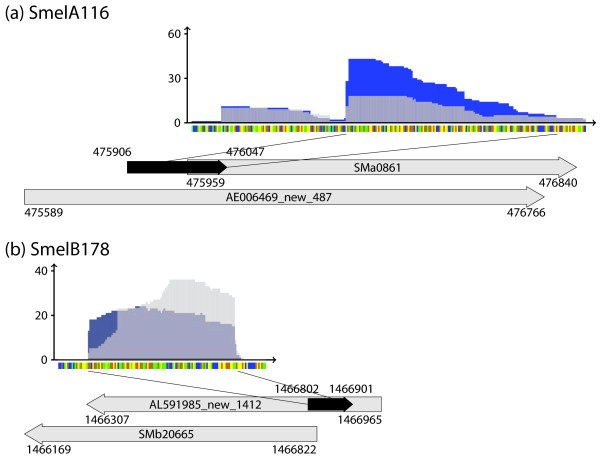
**Examples of sRNAs within transposable elements**. Sequence coverage profile: blue and light grey color denote transcript coverages derived from sample 1 and 2, respectively. Dark grey colored areas represent an overlap of coverages from both samples. y- and x-axis represent coverage and sequence, respectively. Sequence code: blue, A; yellow, C; orange, G; green, U. Grey arrows represent genes flanking or overlapping sRNA genes. Black arrows represent the sRNAs. **(a) **SmelA116 (mRNA leader transcript of SMa0861), **(b) **SmelB178 (antisense transcript of SMb20665).

A number of recent studies revealed non-coding transcripts within transposable elements or repeat regions [9,42]. Hence, the transcriptional activity of these regions in the *S. meliloti *genome [31,32] was analyzed. This suggested repeat regions from two groups showing transcriptional activity. The first group comprises 31 repeat regions, 76 to 166 nt in length, with transcriptional activity [Additional file 3]. These are associated with transposase genes, either in antisense (21 candidates), in sense orientation (9 candidates) or as leader transcript (1 candidate) [Additional file 3]. An example for this group is transposon TRm17 (SMb20665) that occurs in several copies on the chromosome as well as on the megaplasmids. It possesses a type 1 antisense sRNA of 100 nt which overlaps the 5'-end of the transposase gene (Figure 9b). Antisense sRNAs with similar features were found in TRm19 and TRm22. The partial transposases TRm20C and TRm5N are associated with a type 3 antisense sRNA. Furthermore, TRm3, TRm20, SMa1612 and SMa2171 possess sense sRNAs within the transposase-encoding region or overlapping its 3'-end. Interestingly, a putative mRNA leader was identified upstream of SMa0861 which presumably contains two distinct mRNA leader-like transcription start sites (Figure 9a). The second group comprises 26 repetitive extragenic palindromic (REP) elements, 16 repeats and three regions which include both REP elements and repeats in *S. meliloti *[31,43,44] with transcriptional activity [Additional file 3].

### Identification and expression profiling of sRNAs by oligonucleotide microarray and chip hybridizations

As a complementary approach to the deep sequencing strategy, oligonucleotide microarray and Affymetrix Symbiosis Chip hybridizations were carried out using arrays that contain probes representing coding regions and intergenic regions (see "Methods") (data are available in the ArrayExpress database, E-MTAB-204).

RNA for these experiments was obtained from identical stress conditions and growth phases as previously applied for deep sequencing. In the oligonucleotide microarray hybridizations, signals derived from fractions composed of small (< 200 nt) and long (> 200 nt) RNAs were compared (Figure 1c). This strategy allowed for identifying sequence regions predominantly present in the small RNA fraction excluding sequence regions that were also represented in the long RNA fraction at a considerable level. Applying a cut-off of ≥ 8 to the ratio of signals derived from the small RNA to signals from the long RNA fraction 1,906 sRNA candidate regions were identified [Additional file 4]. Among these were the 4.5S RNA, the IncA sRNAs, and a number of tRNAs confirming the applicability of this strategy.

According to the standard procedure of classification (Figure 3), 985 candidates were classified as putative trans-encoded sRNAs (Figure 2c and Table 1). 264 of these were mapped to intergenic regions oriented in antisense to neighboring genes but not overlapping the 5'- or 3'-UTRs and thus classified as type 1 trans-encoded sRNA candidates, whereas 721 were classified as type 2 sharing the same orientation as at least one of the two neighboring genes. The remaining candidates fell into the groups of cis-encoded antisense sRNAs and mRNA leader transcripts (Figure 2c and Table 1).

In support of the oligonucleotide microarray-based analysis, Affymetrix Symbiosis Chip hybridizations were carried out using the small RNA fractions from the experiments described above. Signals were classified as small non-coding RNA candidates when exhibiting the following characteristics: (i) a signal intensity at least two fold higher than the background, and (ii) a distance of less than 150 nt between two probes with a positive signal. Candidates listed in [Additional file 5] were classified following the standard procedure (Figures 2d and 3). Comparison of the microarray with the Affymetrix Symbiosis Chip data revealed 70 trans-encoded sRNAs, 7 cis-encoded antisense sRNAs, and 7 mRNA leader candidates identified by both approaches.

**Figure 10:**
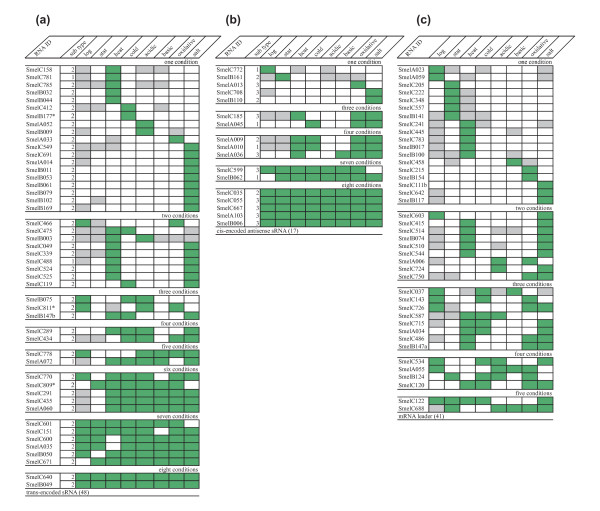
**Expression pattern of sequenced non-coding transcripts**. Expression pattern of (**a**) trans-encoded sRNA, (**b**) cis-encoded antisense sRNAs and (**c**) mRNA leader transcripts identified by deep sequencing: log, stat, heat, cold, acidic, basic, oxidative represent the analyzed stress conditions. Grey, white and green boxes indicate no signal, weak signal (less than 8-fold) and strong signal (≥ 8-fold), respectively. * indicates candidates uniquely identified with Illumina/Solexa sequencing.

The candidates from the oligonucleotide microarray hybridizations were clustered on the basis of their expression profiles under the eight growth conditions tested [Additional file 6]. Information on expression patterns of 48 trans-encoded sRNAs with unambiguous 5'- and 3'-ends identified by deep sequencing could be retrieved from the microarray data (Figure 10). Expression patterns could also be deduced for 17 cis-encoded antisense sRNAs and 41 mRNA leader sequences that are also shared by both data sets (Figure 10). Only 2 of these trans-encoded sRNA candidates appeared to be generally expressed in all conditions. The other candidates from this sRNA class were detected only in a subset of conditions with the largest cluster comprising 19 candidates only found in a single condition. Clustering of the expression patterns of the putative cis-encoded antisense sRNAs shared by both data sets revealed 5 candidates expressed in all conditions, whereas 7 candidates showed expression in two to seven conditions, and 5 candidates in only one condition. From the putative mRNA leader transcripts identified by both approaches, 23 were predominantly detected in the small RNA fraction in two to five conditions, whereas 18 were identified in only one condition. None of these leader transcripts was detected in all conditions.

### Validation of selected candidates

#### Prediction of peptide-encoding sRNAs

Since a short transcript may have dual functions as regulatory sRNA and mRNA [45], sRNA candidate sequences were screened for coding regions of at least 60 nt preceded by a putative ribosome binding site (RBS). Results of this analysis are summarized in [Additional file 7]. Among the trans-encoded sRNAs, six candidates carry such a coding region, of which three are preceded by a predicted RBS. The class of antisense sRNAs includes 6 candidates with a coding region, of which one possesses a putative RBS. In case of sense sRNAs, 24 candidates were found that may encode a peptide and 5 of these also carried an upstream RBS motif. Different types of coding regions were found, ORFs that overlap the corresponding gene or are located within the coding region of this gene. The reading frame of most of the predicted sRNA ORFs differs from the reading frame of the corresponding gene, but 10 share the same reading frame and hence the same stop codon with this gene. A similar situation was found in the class of mRNA leader transcripts. Here, 49 candidates include an ORF, 8 of these with a potential RBS. These ORFs do not include those starting with the start codon of the corresponding gene because these lack an inframe stop codon on the sRNA sequence. Only ORFs were included that map to the 5'-UTR or overlap the coding region of the corresponding gene in a different reading frame.

### Analysis of sRNA candidates by 5'-RACE and Northern hybridizations

**Figure 11:**
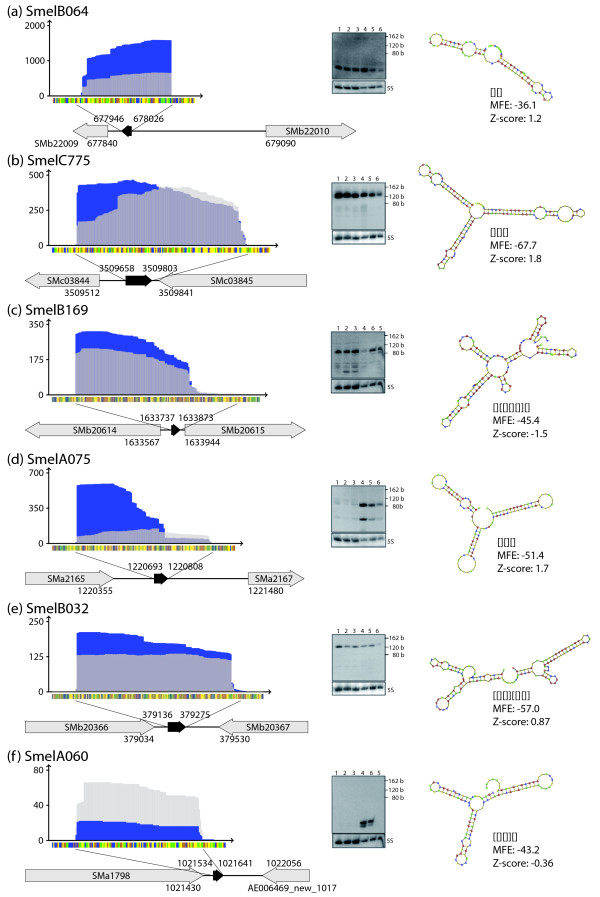
**sRNA candidates validated by Northern hybridizations and 5'-RACE**. Sequence coverage profile: blue and light grey color denote transcript coverages derived from sample 1 and 2, respectively. Dark grey colored areas represent an overlap of coverages from both samples. y- and x-axis represent coverage and sequence, respectively. Sequence code: blue, A; yellow, C; orange, G; green, U. Grey arrows represent genes flanking or overlapping sRNA genes. Black arrows represent the sRNAs. MFE: minimum free energy within the shape class. Validated by Northern hybridizations: trans-encoded sRNAs SmelB064 **(a) **and SmelC775 **(b)**. Validated by 5'-RACE: trans-encoded sRNAs SmelB169 **(c)**, SmelA075 **(d)**, SmelB032 **(e)**, SmelA060 (two copies in the genome, second copy SmelA072) **(f)**. Lanes: 1, TY (control for cold shock); 2, cold shock; 3, TY (control for heat shock); 4, heat shock; 5, GMX (control for salt chock); 6, salt shock.

**Figure 12:**
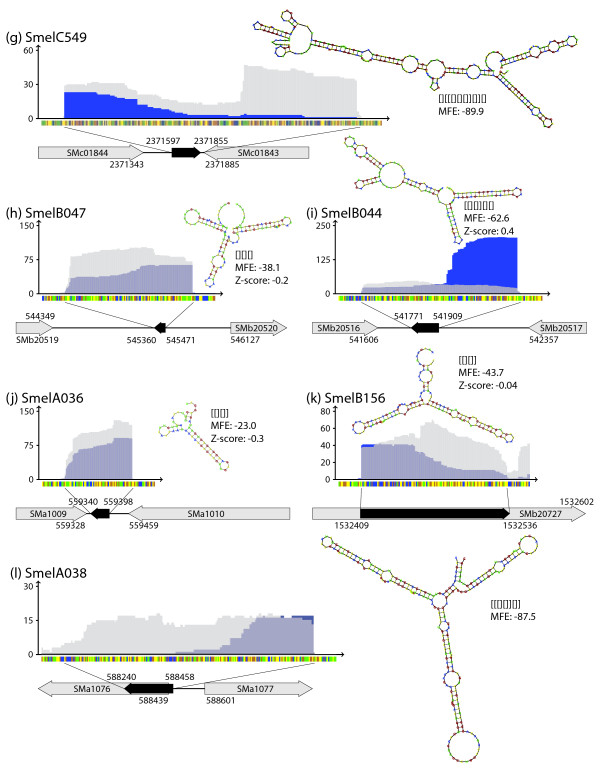
**sRNA candidates validated by 5'-RACE**. Sequence coverage profile, grey arrows, black arrows: see legend to Figure 11. Validated by 5'-RACE: trans-encoded sRNAs SmelC549 **(g)**, SmelB047 **(h)**, SmelB044 **(i)**, antisense sRNA SmelA036 **(j)**, sense sRNA SmelB156 **(k)**, and mRNA leader SmelA038 **(l)**. Lanes: see legend to Figure 11.

sRNA candidates selected from different classes were further analyzed by 5'-RACE and Northern hybridizations (Figures 11 and 12). RACE experiments confirmed the 5'-ends derived from the 454 sequencing data of the trans-encoded sRNAs SmelB169, SmelA075, SmelB032, SmelC549, SmelB047, and SmelB044, the cis-encoded antisense sRNA SmelA036, the mRNA leader transcript SmelA038, and the intragenic sense sRNA SmelB156.

Northern hybridizations were carried out for six candidates (Figures 11 and 12) using total RNA obtained from the exponential growth phase in complex medium, after shifting the culture to higher or lower temperature, and after adding salt to the culture. Hybridizations of the trans-encoded sRNAs SmelB064, SmelC775, SmelB169, and SmelB032 showed signals in all conditions with just small variations in signal strength. In contrast, SmelA075 and SmelA060/SmelA072 showed differential expression. SmelA075 was only detected at a very low level in complex medium and in the same medium at decreased temperature. However, it was strongly detected after the shift from 30°C to 40°C and at a medium level in GMS medium. SmelA060 and SmelA072 encode two sRNAs of the same length that differ by just one nucleotide. These sRNAs were detected only after the temperature shift to 40°C and after adding 400 mM NaCl to a culture in GMS medium. Microarray hybridization data is available for SmelA060/SmelA072 represented by oligonucleotide probes on the array. These results are in good agreement with the results of the Northern hybridization, with the highest ratios obtained under heat shock (73-fold) and salt shock conditions (17-fold), and values slightly above the threshold (8-fold) for sRNA detection in the other conditions. This indicates that expression patterns can be retrieved from the microarray data.

The sRNA lengths of SmelB064 and SmelB169 (TPP riboswitch) estimated from the Northern hybridizations are shorter than those derived from the sequencing data. This might be explained by incomplete denaturation of these sRNAs or by degradation processes during RNA isolation. In case of SmelB169 a weak band at the expected size was detected that may correspond to the full length contig derived from the sequencing data. Northern hybridization of Smel064 resulted in two close bands which may have originated from alternative 3'-ends found by cDNA sequencing. Two dominant bands were also detected for SmelA075 and SmelA060/SmelA072. The former are also indicated by the sequence coverage (Figures 11 and 12) whereas no hint on the origin of the additional band could be found for SmelA060/SmelA072. Interestingly, a structure composed of three stem-loops with identical sequence motifs in the loops was predicted for SmelA075 (Figure 11D). Shorter transcripts suggested by Northern hybridizations and cDNA sequencing were predicted to lack one or two of the stem-loop substructures [Additional file 8]. Stable structures corresponding to predicted structural domains of the full-length transcripts were also predicted for shorter transcripts associated to the sRNAs SmelC549, SmelB044, and SmelB156 (Figure 12g, i, and 12k) [Additional file 8]. This is not the case for the putative mRNA leader transcript SmelA038. The structure predicted for the short 5'-transcript differs from that of the full-length RNA (Figure 12) [Additional file 8].

**Figure 13:**
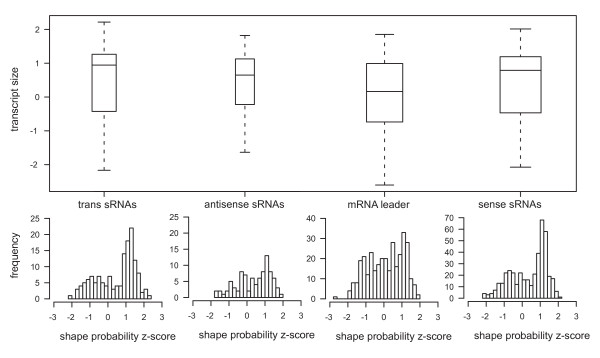
**Z score-distribution**. Distribution of Z-scores for dominant shape probabilities in different classes of transcripts. Shape probabilities serve as a measure of the well-definedness of secondary structure, which is independent of GC content. See Methods for details of the Z-score computation. The same data are shown as box plots (indicating median, first quartiles and extremal points) and as histograms. A bias towards positive Z-scores, strongest for trans-encoded sRNAs, and almost zero for mRNA leader transcripts is seen.

Z-scores computed for the sRNA candidates (see structural analysis below) significantly differ from zero indicating that these sRNAs form a well-defined secondary structure (Figure 13). Predicted structures and Z-scores of the sRNAs analyzed by Northern and 5'-RACE are shown in Figures 11 and 12.

### Bioinformatics analysis of sRNA candidates

#### BLAST Homology Search

BLAST homology searches for the 1,080 transcripts from 454 sequencing were carried out at different cut-offs. We report here on results for an E-value cut-off at 7e-15, which is the most stringent cut-off for which all our transcripts match against their origin genome. The match covering at least 70% of the transcript length was an additional requirement. Data for this setting is shown in Table 3. Under these criteria, we obtained about 9,000 hits in alpha-proteobacteria, and about 5,000 in other bacteria. Hits were classified as "known" if there was annotation information associated with the matched region. As expected, the class of sense transcripts comprised the highest percentage of "known" matches (44%), followed by mRNA leaders (15%). The low percentage for antisense and trans-encoded transcripts (both 6%) can be explained by the fact that only few transcripts of this type are annotated in the databases. Data for other BLAST cut-offs are given in [Additional file 9].

**Table 3:** Blast search results

E-value 7e-15 und target length >= 0.7 * query length						
tax_group	known	cis-encoded antisense sRNA	cis-encoded mRNA leader	ORF	sense sRNA	trans-encoded sRNA
*Sinorhizobium meliloti*	n	103	385	8	462	203
						
*Sinorhizobium meliloti*	n	2	38	1	75	13
*Sinorhizobium meliloti*	y	7	39	0	134	15
*Sinorhizobium*	n	52	306	7	332	94
*Sinorhizobium*	y	2	7	0	295	0
*Sinorhizobium*/EnsiferGroup	n	0	0	0	0	0
*Sinorhizobium*/EnsiferGroup	y	0	0	0	72	0
*Rhizobiaceae*	n	105	604	15	1099	191
*Rhizobiaceae*	y	1	42	1	915	18
*Rhizobiales*	n	104	290	4	1538	242
*Rhizobiales*	y	6	145	0	1433	3
Alphaproteobacteria	n	10	47	2	696	9
Alphaproteobacteria	y	1	0	0	39	0
						
		0.059	0.15	0.03	0.44	0.061
						
Gammaproteobacteria	n	0	30	0	726	0
Gammaproteobacteria	y	0	0	0	225	0
Betaproteobacteria	n	6	27	3	881	0
Betaproteobacteria	y	0	0	0	556	0
delta/epsilonSubdivisions	n	0	3	0	228	0
delta/epsilonSubdivisions	y	0	0	0	212	0
unclassified Proteobacteria	n	0	0	0	2	0
unclassified Proteobacteria	y	0	0	0	1	0
Bacteria	n	0	6	0	610	0
Bacteria	y	0	0	0	442	0
other	n	1	6	0	105	3
other	y	1	32	0	592	3

Results of the BLAST search with sRNA trancripts against sequence databases with an E-value cut-off 7e-15 and the requirement that the hit covers at least 70% of the query are shown. Hits are classified as known (marked y in the "known"column) if annotation information is associated with the database sequence. Hit counts are given in taxonomic order. Hits to the taxonomic group do not include the hits to the subgroup which is listed above it. For the alpha-proteobacteria (including all subgroups), the percentage of "known" versus "unknown" hits for each class of transcript is listed. y, known; n, unknown.

#### Structural analysis

We investigated whether our transcripts appear to have a well-defined secondary structure. Rather than looking at individual minimum free energy foldings, we computed accumulated Boltzmann probabilities of all (optimal or suboptimal) foldings of the RNA which exhibit the same abstract shape [46]. We determined the dominant shape for each sequence and computed Z-scores against a background distribution taken from the *S. meliloti *genome [Additional file 10] (see Methods for details of the Z-score computation). The result is shown in Figure 13, which shows that classes of trans-encoded, sense and antisense trancripts tend to have positive Z-scores, centered around +1. This implies that for these classes a well-defined secondary structure may be associated with their function. Leader transcripts showed no bias.

#### Standard Rfam homology search

From the 31 non-coding sRNAs from *S. meliloti *already known in Rfam, belonging to 16 families, our approach confirmed 21 by direct positional matching, although with corrections in their precise position. These were also returned by standard Rfam search with regular cut-offs [Additional file 11]. Beyond these known transcripts, 16 candidates were classified as - heretofore unknown - members of 13 different RNA families, excluding three families of HIV sequences attracting 56 hits, which were considered false positives. 11 of the remaining families included known members from *S. meliloti*. The only "new" families resulting from this search were RF00037 (iron response element) and RF00556 (HBV RNA encapsidation signal epsilon), the latter of which appears biologically implausible. Hence, members of only one new family were identified applying a standard Rfam search.

#### Refined Rfam homology search

We performed a simple but complete evaluation of the family models presently available in Rfam, comparing scores obtained from the covariance models to scores from plain sequence models generated from the same sequence alignments (data not shown). We observed that most of the covariance models strongly rely on sequence similarity. They practically behave as HMM sequence models, with the encoded structure only contributing to the score in a negligible way. A diverged sequence with a conserved structure will rarely pass the model thresholds, explaining the lack of generalization.

As known sRNAs are scarce in the class of alpha-proteobacteria, we sought a way to extract more information from Rfam models. We designed a pipeline using *RNAsifter *[47] that combines independent evidence of structural conservation with an Rfam search below the suggested cut-offs (see "Methods"). By this procedure, we obtained (after filtering human, viral, and microRNA sequences) 33 families and 97 families above cut-offs of 0.5*T and 0.25*T, respectively, where T is the default cut-off of the family. These candidates require individual analysis; an encouraging observation about the refined search strategy is the correct classification of SmelA062 as a group II intron, which was neither observed with the standard Rfam search nor applying a BLAST search. The list of new families is given in [Additional file 11].

## Discussion

### Identification of sRNA candidates in *S. meliloti*

Here we report on the first comprehensive screen for sRNAs in an alpha-proteobacterium applying deep sequencing and microarray hybridizations. The symbiotic nitrogen-fixing soil bacterium *S. meliloti *was analyzed as representative of the *Rhizobiales*. Our study suggested a total of 1,125 sRNA candidates that were classified as trans-encoded sRNAs (173), cis-encoded antisense sRNAs (117), mRNA leader transcripts (379), and sense sRNAs overlapping coding regions (456). These results are in good agreement with a number of recent studies that reported genome-wide screens for prokaryotic sRNAs by cDNA sequencing and hybridization to tiling microarrays [28]. The first studies of this type were reported for *E. coli*, but similar studies were also published for other Gram-negative bacteria (e.g. *Salmonella typhimurium Prochlorococcus marinus*, *Vibrio cholerae*, *Aquifex aeolicus*, *Pseudomonas aeruginosa*, and *Burkholderia cenocepacia*) and Gram-positive bacteria (e.g. *Bacillus subtilis*, *Bacillus anthracis*, *Listeria monocytogenes*, and *Streptomyces coelicolor*). In the group of alpha-proteobacteria, only one global sRNA screening study was published so far, which was a tiling microarray-based sRNA screen in *C. crescentus *[29] that resulted in 27 novel sRNA candidates. For alpha-proteobacteria, only one pilot study applying pyrosequencing of cDNA derived from total RNA depleted for rRNA sequences of *S. meliloti *was reported [40]. But this approach aimed at identifying transcripts of new protein-encoding genes and delivered just 1,913 reads derived from non-rRNA sequences that indicated 20 new ORFs. Depending on the methods and strategies of data analysis applied, and the selection criteria for sRNA candidates, these studies suggested up to several hundred sRNA candidates in different bacteria. This indicates that not only miRNA genes in eukaryotes but also sRNA genes in bacteria represent a significant proportion of the genome, although only a small proportion of these candidates was further validated.

#### sRNA discovery by deep sequencing

For discovery of sRNAs in *S. meliloti *we have combined two deep sequencing technologies. 454 sequencing of cDNAs turned out to be the most valuable data resource. Sequence coverage defined 5'- and 3'-ends of the sRNA candidates which are of great advantage for both structural and comparative analyses. Moreover, enrichment of primary 5'-ends of transcripts allowed for identifying transcriptional start sites of sRNA candidates with several of these validated by 5'-RACE. Data from enrichment of processed 5'-ends was ambiguous with a very high proportion of 5'-ends present in both the primary and the processed transcript-enriched samples. This effect may have been caused by a pyrophosphatase activity which eliminates the pyrophosphate from the 5'-end of primary transcripts making these transcripts accessible for the sequencing procedure followed in the processed transcript-enriched sample. Pyrophosphatase activity was recently described in several bacteria, such as the Nudix enzymes *RppH *in *E. coli *and *BdRppH *in *Bdellovibrio bacteriovorus *[48-51]. Nevertheless, a number of sRNA candidate regions displayed alternative 5'-ends that may have been caused by posttranscriptional processing of the transcripts. Processing of transcripts at the 5'-end is a well characterized mechanism, an example of which is the sRNA *GadY *in *E. coli *[21]. We also found a number of sRNAs with transcription start sites varying by only one or two nucleotides. These were probably generated by the activity of a single promoter with a diffuse transcription start site, as was previously described for the *SraL *sRNA [52]. Alternatively, transcripts with more than one distinct 5'-end may be products of alternative promoters.

Alternative 3'-ends observed in this study might be generated by endo- or exonucleolytic cleavage of the full length transcript. Examples of sRNAs processed by such mechanisms are MicX in *Vibrio cholerae *[53] and ArcZ (*sraH*) in *E. coli *and *Salmonella *[52,54]. The former is processed by RNAse E into two transcripts with different 3'-ends whereas the primary and 5'-processed transcripts of the latter undergo 3'-exonucleolytic degradation. Another possible mechanism that results in alternative 3'-ends is the presence of two or more termination sites. For instance, *gcvB *of *E. coli *possesses two termination sites, resulting in two distinct transcript sizes [52,55]. Illumina/Solexa sequencing of cDNA derived from total RNA revealed only few additional trans-encoded sRNA candidates.

The deep sequencing data from this study suggests an average length of the sRNA candidates of about 120 nt. This is in good agreement with results from several other studies that deduced a typical size range of sRNAs of 50 to 300 nt [2,56].

#### Filtering and classification of sRNA candidates

The stringency of criteria for selection of sRNA candidate regions based on cDNA sequencing data has a great effect on the number of candidates derived from this approach. In this study, we have based the 454 sequence data analyses on strict criteria that defined seed regions of 50 to 350 nt in length with a coverage of at least 10 reads per nucleotide. Selection criteria were relaxed to a minimum of five reads per nucleotide to complete the contigs. Definition of a lower coverage would have resulted in a higher number of candidate regions with an increasing number of false positives from transcriptional background and mRNA degradation.

Classification of sRNA candidates in putative trans-encoded sRNAs, cis-encoded antisense sRNAs, mRNA leader transcripts, and sense sRNAs overlapping coding regions is ambiguous. The main difficulty is the definition of a gene region including 5'- and 3'-UTR, since promoter and terminator predictions are not well established in *S. meliloti*. Therefore, the classification was based on the estimated minimal length of 5'- and 3'-UTRs which might have resulted in misclassification of a number of candidates. Moreover, the majority of sense sRNAs completely or partially overlapping coding regions probably are stable mRNA degradation products.

Several previous studies provided also evidences for the presence of antisense sRNA and mRNA leader transcripts in addition to the intergenic trans-encoded sRNAs [57]. Cis-encoded antisense sRNA transcripts appear to be very dominant in *Synechocystis *sp. PCC 6803. Georg *et al*. [58] suggested that about 10% of all genes in this organism are influenced by antisense RNAs. Recently, evidence for 127 antisense RNAs that may affect about 3% of the protein-encoding genes was obtained by parallel sequencing in *Vibrio cholerae *[57]. Such a high proportion of antisense transcripts as in *Synechocystis *sp. PCC 6803 was not observed in our study in *S. meliloti *applying deep sequencing. Only about 2% of the protein-encoding genes seem to be partly transcribed in antisense direction. In contrast, our microarray experiments suggested about 11% of genes with overlapping antisense transcripts. Such discrepancy may be organism-specific, but probably is more influenced by employing different discovery strategies.

In this study, a multi-tiered approach to sRNA identification was taken applying different complementary identification strategies. Illumina/Solexa sequencing was carried out for cDNA derived from total RNA which resulted in a proportion of 87% of reads mapping to rRNA genes or repeat regions. Usage of total RNA and the inability to retrieve strand information from this sequencing data did not allow for identifying sRNAs associated to coding regions, but identified intergenic sRNA genes and revealed information on transcription units of protein-encoding genes (data not shown). In contrast, the 454 sequencing approach including size fractionation and primary 5'-end enrichment provided rich information on short transcripts encoded in intergenic regions as well as antisense sRNAs and leader transcripts associated to coding regions.

A microarray approach cannot provide exact information on 5'- and 3'-ends of sRNAs and is limited by the genome coverage with oligonucleotide probes. The Sm14kOLI microarray used in this study contained probes distributed on both strands of intergenic regions at irregular distances. Coding regions were just represented by a single probe. The strategy of comparing short RNA to long RNA fractions was therefore tailored to predominantly identify intergenic sRNA genes. In comparison to the high number of microarray hybridizations covering different conditions, only a low number of hybridizations of short RNA to the Affymetrix Symbiosis Chip were performed resulting in only few reliable sRNA candidates.

**Figure 14:**
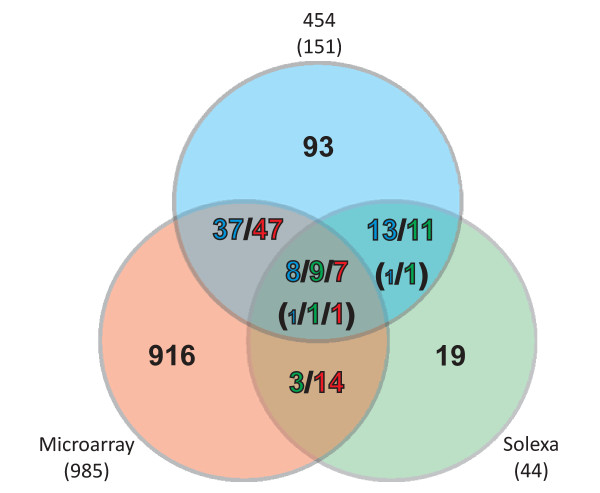
**Venn diagram comparing trans-encoded sRNA candidates identified by 454 sequencing, Illumina/Solexa sequencing, and microarray hybridizations**. In some cases a sRNA region detected by one method overlaps with multiple regions detected by one or both of the other methods. This is indictated by the colors of the numbers in the fields representing the overlaps. Numbers in brackets indicate discrepancies in the classification of sRNA regions identified by different methods. Small numbers indicate 454 deep sequencing candidates not classified as trans-encoded sRNA.

Because of the different strengths of the applied strategies, a reasonable comparison of identified candidates is only possible for the class of trans-encoded sRNAs (Figure 14). About 40% of the trans-encoded sRNAs identified by the 454 sequencing approach were also identified by the Illumina/Solexa sequencing and/or the microarray approaches. Only 9 candidates were identified by all approaches. It has to be noted that these numbers change with relaxing the strict criteria applied in analysis of the deep sequencing data. Classification and determination of sRNA ends is more reliable based on the 454 sequencing-derived dataset. Because of the short Illumina/Solexa reads as well as distribution and length of microarray probes, the definition of the regions is less accurate based on these approaches. This explains discrepancies in classification of a few sRNA regions and mapping of some sRNA regions identified by one method to multiple regions identified by one or both of the other approaches.

### Deep sequencing data confirmed previously reported sRNAs

In addition to 4.5S RNA, tmRNA, tRNAs and rRNAs, our deep sequencing approach confirmed 83 previously published sRNA candidates. The tmRNA [33], the two IncA antisense transcripts [12], and two ROSE-like elements (Repression Of heat-Shock gene Expression) [25] were found in studies dedicated to one or few sRNAs, whereas the other candidates resulted from global screening approaches [33,37-39] (Table 4).

**Table 4:** sRNA candidates identified in previous studies

ID	Type	Position	St.	Size	Rfam	St.	Ov.	**ID **[36]	St.	Ov.	**ID **[37]	St.	**ID **[38]	St.	Ov.	North.
SmelC011	3	46011-46166	-	156							sra02	n				
SmelC023	1	201679-201825	+	147				C7	+	100	sra03	+	SmrC7	+	98	[37-39]
SmelC035	2	259927-260032	+	106	RF00169	+	97				sra05	+				
SmelC037	3	267059-267152	+	94									Sm76	+	59	[39]
SmelC051	3	318407-318497	+	91							sra09	n				
SmelC057	4	364136-364231	-	96									Sm134	+	32	
SmelC073	1	424110-424268	+	159									Sm131	+	72	
SmelC089	3	523807-524015	+	209									Sm39	+	33	
SmelC100	3	580087-580184	-	98	RF00521	-	76				sra13	n				
SmelC116	2	658552-658723	-	172									Sm105	-	49	
SmelC119	1	701950-702109	+	160							sra16	-				
SmelC132	3	757653-757739	+	87									Sm139	+	100	
SmelC213	2	1048676-1048864	+	189							sra25	+				[38,39]
SmelC222	3	1071388-1071540	-	153									Sm145	-	67	[39]
SmelC226	1	1091100-1091291	+	192									Sm10	+	100	
SmelC246	3	1238618-1238804	-	187							sra29	-				[38]
SmelC289	1	1398278-1398426	-	149				C9	-	81	sra32	-	SmrC9	-	99	[37-39]
SmelC291	1	1411678-1411848	+	171				C10	+	77	sra33	+				[38]
SmelC302	3	1458322-1458424	+	103							sra34	-				[38]
SmelC306	4	1461414-1461561	+	148							sra35	+				
SmelC311	3	1464122-1464318	+	197							sra36	+				
SmelC337	1	1518791-1518985	+	195									Sm30	+	100	
SmelC365	4	1599106-1599255	+	150									Sm55	-	24	
SmelC378	4	1635062-1635243	+	182									Sm9	-	15	
SmelC381	1	1637121-1637244	+	124							sra37	-	Sm50	-	48	
SmelC397	1	1667491-1667614	-	124				C14	-	100			SmrC14	-	100	[37]
SmelC398	1	1667766-1667982	-	217							sra38	n	Sm7	-	99	
SmelC411	1	1698618-1698731	-	114	RF00519	-	62	C15	-	100			SmrC15	-	100	[37]
SmelC412	1	1698818-1698948	-	131	RF00519	-	57	C16	-	100	sra41	n	SmrC16	-	98	[37,38]
SmelC414	3	1706675-1706765	-	91									Sm23	-	56	
SmelC416	1	1718814-1718919	-	106									Sm138	-	85	
SmelC419	5	1728029-1728277	-	249									Sm6	-	97	
SmelC434	1	1821211-1821366	+	156									Sm118	+	100	
SmelC435	1	1823103-1823231	+	129									Sm48	+	100	
SmelC445	3	1879868-1879947	-	80							sra44	n				
SmelC475	1	2059726-2059795	-	70									Sm29	-	100	
SmelC483	1	2098461-2098583	-	123				C17	+	100						
SmelC488	1	2129185-2129292	-	108									Sm135	-	99	
SmelC525	1	2291226-2291466	+	241							sra49	+				
SmelC531	2	2321050-2321287	-	238									Sm26	-	98	[39]
SmelC549	1	2371597-2371855	+	259									Sm4	+	99	
SmelC559	4	2398177-2398336	+	160	RF00050	+	100	C20	+	91						
SmelC561	4	2436481-2436577	+	97									Sm52	-	100	
SmelC576	4	2524155-2524249	+	95									Sm136	-	27	
SmelC587	3	2575832-2575947	-	116									Sm104	-	67	
SmelC617	1	2695496-2695642	-	147									Sm49	-	100	
SmelC642	3	2921789-2922084	+	296							sra54	n				
SmelC646	3	2924467-2924555	-	89							sra55	n				
SmelC653	3	2937985-2938127	-	143	RF00517	-	34									
SmelC667	2	2972090-2972252	-	163	RF00013	-	98	C22	-	83	sra56	-				[37-39]
SmelC671	1	2986421-2986520	+	100									Sm84	+	100	[39]
SmelC689	1	3046710-3046789	+	80	RF00519	+	81						Sm8	+	96	[39]
SmelC691	1	3048822-3048904	+	83							sra57	n				
SmelC706	1	3105298-3105445	-	148	RF00518	-	100	C45	-	52						[37]
SmelC752	3	3439569-3439771	+	203							sra67	+				
SmelC762	3	3461772-3461886	+	115	RF00521	+	63									
SmelC764	4	3472924-3473053	+	130	RF00519	+	44									
SmelC778	1	3522269-3522383	+	115									Sm5	+	97	
SmelC779	3	3532867-3532968	+	102	RF00059	+	100									
SmelC804*	1	1599291-1599363		73									Sm55	-	73	
SmelC805*	1	1677330-1677472		143							sra40	n				
SmelC809*	1	2356793-2357134		342	RF00010	-	100	C19	-	100	sra50	+				
SmelA033	1	512140-512221	-	82				A2	+	100			smA3a	-	83	
SmelA035	1	552854-552983	+	130	RF00519	+	55						smA4b	+	98	
SmelA075	1	1220693-1220808	+	116									smA8	+	99	
SmelA099	1	1328175-1328334	-	160				A6	+	100						
SmelA102	3	1351156-1351309	-	154	RF00490	-	43									
SmelA103	2	1351298-1351357	+	60	RF00489	+	73									
SmelB001	1	24911-24992	-	82				B29	+	100						
SmelB006	2	56486-56620	+	135	RF00489	+	35	B30	+	52						
SmelB011	1	66261-66326	+	66	RF00174	+	100	B31	+	21						
SmelB019	1	213497-213616	+	120									smB1	+	93	
SmelB027	2	334403-334483	-	81				B34	+	100						
SmelB032	1	379136-379275	+	140									smB2	+	91	
SmelB044	1	541771-541909	-	139									smB3b	-	100	
SmelB050	1	574628-574763	+	136									smB5a	+	100	
SmelB053	1	577730-577873	+	144				B35	+	99			smrB35	+	97	[37]
SmelB085	3	871959-872147	-	189	RF00435	-	50									
SmelB110	2	1192051-1192144	-	94	RF00059	-	100									
SmelB126	1	1325476-1325586	+	111									smB9	+	100	
SmelB169	1	1633737-1633873	+	137	RF00059	+	80									

Overlap, percent overlap of the candidates from the cited studies or database compared to the region covered by a sRNA candidate in this study. n, strand was not or not undoubtedly determined.

ID; RNA ID (this study); Type, RNA type; St., strand; Ov., overlap (%); ID [], RNA ID corresponding to the given reference; North., experimentally validated by Northern blot, given are the references for the Northern hybridizations. RNA ID: *, Solexa candidate. RNA type: 1, trans-encoded sRNA; 2, cis-encoded antisense sRNA; 3, mRNA leader; 4, sense sRNA; 5, Open reading frame. The presumed location of the sRNAs predicted by Ulvé *et al*. [37], derived from the location of the Dot Blot probes. Thus an overlap analysis to our candidates could not be performed.

**Figure 15:**
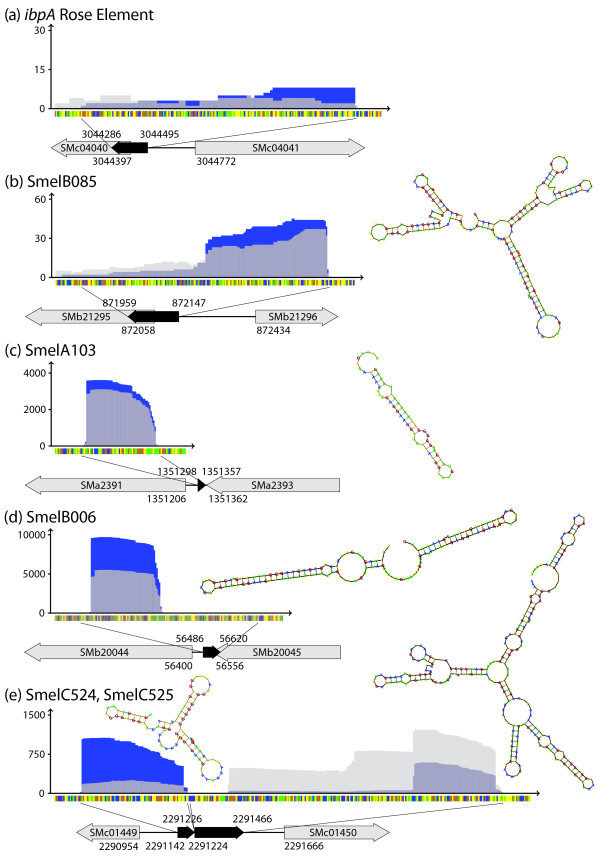
**ROSE elements, IncA antisense RNAs, and tmRNA identified by 454 sequencing**. Sequence coverage profile: blue and light grey color denote transcript coverages derived from sample 1 and 2, respectively. Dark grey colored areas represent an overlap of coverages from both samples. y- and x-axis represent coverage and sequence, respectively. Sequence code: blue, A; yellow, C; orange, G; green, U. Grey arrows represent genes flanking or overlapping sRNA genes. Black arrows represent the sRNAs. **(a) ***ibpA *ROSE element (no ID), **(b) **SMb21295 ROSE element (SmelB085), **(c) ***incA *located on *pSymA *(SmelA103), **(d) ***incA *located on *pSymB *(SmelB006). (**e**) both fragments, SmelC524 and SmelC525 of the tmRNA.

Among the mRNA leader transcripts were two candidates (Figure 15a and 15b) that corresponded to the predicted ROSE-like elements upstream of *ibpA *and SMb21295 encoding heat shock proteins in *S. meliloti *[25]. It has to be noted that the *ibpA *ROSE element was represented by only eight reads (rather than ten) in the sequencing data and thus marginally failed our filter for candidate selection.

MacLellan *et al*. [12] characterized the cis-encoded antisense sRNA gene *incA *situated within the *repABC *operon on each megaplasmid. *incA *is located within the small intergenic region between *repB *and *repC *in opposite orientation to these coding regions. High sequence coverage of both IncA sRNAs was also evident in the sequencing data (Figure 15c and 15d).

The *S. meliloti *tmRNA (SsrA) was identified because of its homology to Sra from *Bradyrhizobium japonicum *[33]. Northern blot analysis and mapping of both 5'- and 3'-ends identified two pieces, a 214 nt mRNA-like domain and a 82 nt tRNA-like domain, both highly stable, whereas the premature form was unstable. Both transcripts were also identified in our study with a high 454 sequence coverage (Table 4, Figure 15e).

The *S. meliloti *strain used in this study harbours three classes of group II introns, RmInt1 (bacterial class D), SMb21477/SMb21167 (bacterial class C), and SMa1875 (unclassified) [59]. Transcript SmelA062 probably corresponding to the intron lariat RNA was identified by the 454 sequencing approach.

Our sequencing data supported 73 sRNA candidates that resulted from the previous genome-wide screens of which 17 were confirmed by Northern hybridizations [37-39] (Table 4). From the 173 trans-encoded sRNAs identified in our study, 42 have previously been predicted (Table 4). This high percentage of re-discovery of known sRNA candidates increases the confidence in the 131 new putative sRNAs. In the classes of antisense sRNAs, mRNA leader transcripts, and sense sRNAs, only few known candidates were re-identified. These were 9 out of 96 antisense sRNAs, 21 out of 378 mRNA leader transcripts, and 8 out of 447 sense sRNAs. Hence, our approach resulted in a high number of new candidates. Furthermore, additional information on 5'- and 3'-ends as well as expression patterns under several growth and stress conditions could be derived for a number of previously described candidates from the sequencing and microarray data. In several cases our sRNA regions differed in orientation from, only partially overlapped, or were located inside the regions predicted in other studies (Table 4). Our approach also confirmed 21 Rfam annotated transcripts of *S. meliloti *1021 (Table 4).

### Putative sRNA functions in *S. meliloti*

#### Putative functions of sRNAs

Trans-encoded and cis-encoded antisense sRNAs predominantly seem to act via base pairing mechanisms [42]. The former usually are located in intergenic regions and share only poor and incomplete complementarity with target sequences, which complicates computational target predictions. In contrast, the latter share extended regions of complementarity to an overlapping sense encoded gene. Thus, in most cases antisense sRNAs modulate expression of the overlapping sense gene on transcriptional or posttranscriptional level, although trans targets may also be affected [42,56].

The majority of antisense sRNAs identified in this study are complementary to the 3'-UTR of the corresponding gene. It is well established that this type of antisense RNAs is involved in posttranscriptional regulation of the steady-state amount. Thus, either mRNA stabilization by masking of potential ribonuclease recognition sites or mRNA degradation by formation of a ribonuclease sensitive RNA duplex (e.g. GadY and RyhB in *E. coli*) [20,21] are probably mediated by the 50 antisense sRNAs mapping to the 3'-UTR. Only 4 cis-encoded antisense sRNAs were located in small intergenic regions in antisense to both neighboring genes which presumably form an operon structure. This type of sRNA may mediate transcription termination inside the operon structure resulting in down-regulation of the downstream gene expression [42].

Microarray hybridizations supported a number of sRNA candidates identified by the deep sequencing approach and provided further information on expression patterns in different growth phases and stress conditions. A group of 19 trans-encoded sRNA candidates, 5 antisense sRNAs, and 18 mRNA leader candidates were found only in one of the eight conditions tested. This indicates a specific expression pattern and provides hints to the biological context of their function. Among these were the antisense sRNA SmelA013 found under oxidative stress as well as SmelC708 and SmelB110 found under salt stress conditions. 5 of the mRNA leader candidates were only found in response to heat stress.

The majority of cis-encoded antisense sRNAs have been described in plasmids, phages and transposons [60]. Such sRNAs probably inhibiting translation of the transposase are encoded by Tn10 (IS10) and Tn30 (IS30) [60-62]. Transposon-encoded antisense RNAs are quite stable which may explain the high sequence coverage of the majority of the transposable element-related sRNA candidates found in our study. Another group of repeats that were represented in the small RNA fraction analyzed in this study were REP elements [31,43,44]. Although these elements are widely distributed within the genome, their functions remain obscure. It has been shown that their presence can affect the level of gene expression [63]. It was speculated that these elements play a role in gene transcription termination. Another proposed role is in relation to chromosomal structure, because both DNA gyrase and DNA polymerase I can bind to REP elements [64,65].

#### Functional classification via database search

An important observation from this study is that searching Rfam family models with over 1,000 sRNA candidates only re-discovered those sequences from *S. meliloti *already known and stored in Rfam. There was not a single case of an unclassified transcript that was predicted to be a member of a certain family, except for the IRE family and some apparent false positives. This is in strong contrast to the state of the art with protein-coding genes. It can be explained in part by the fact that identification of sRNAs in bacteria is still at its outset, and in part by the fact that current Rfam models are very strongly based on sequence similarity rather than conserved structure. Alternative ways to construct structural family models have been recently suggested [66], but an implementation is not yet available. Our refined Rfam search strategy yielded family associations which are still awaiting validation. The BLAST search suggested a number of homologous regions, mainly in the *Rhizobiales *but also in other alpha-proteobacteria, indicating that sRNA candidates derived from the comprehensive experimental screen in *S. meliloti *may support the computational prediction of sRNAs in related bacteria.

## Conclusions

### Genome-wide experimental screening for sRNAs

Several genome-wide screens for sRNAs applying parallel sequencing of cDNAs and hybridizations of tiling microarrays have recently been published. Apart from a single study that used tiling oligonucleotide chips to discover sRNAs in *C. crescentus*, our study is the first comprehensive approach to discover sRNAs in an alpha-proteobacterium. Therefore, this data set will be a valuable resource for comparative studies in related bacteria.

### Identification of homologous sRNAs and assignment to RNA families

Assigning sRNA candidates to functional RNA families with present bioinformatics tools is a difficult task. A recent study [67] shows that even human expertise in describing structural patterns does not take us much beyond a simple BLAST search. The study argues for an effort in developing curated databases of aligned secondary structures, as a prerequisite for increasing the power of comparative analysis. Another perspective is that the use of structural models from Locomotif [68], which include the thermodynamics of RNA folding and can handle simple cases of pseudoknots, may generalize better than the combinatorial pattern descriptions via RNAMotif [69] used in the cited study.

### Perspectives for functional analyses

Global experimental screening strategies have delivered a high number of sRNA candidates in all bacteria studied so far. In contrast to these well-developed discovery strategies, approaches to identify targets of sRNAs in high throughput are still in the infancy. Experimental approaches mainly rely on transcript or protein profiling in sRNA mutant or over-expression strains, and *in vitro *analysis of sRNA-mRNA interaction. In contrast to computational predictions of eukaryotic microRNA targets, predictions of bacterial sRNA targets are complicated by poor and incomplete complementarity. A first small step towards unravelling the functions of the sRNAs in *S. meliloti *is provided by the expression patterns of a subset of sRNAs derived from this study.

## Methods

### Cultivation of *S. meliloti *strain 2011

Pre-cultures of *S. meliloti *strain 2011 [70] were grown at 30°C in TY [71] or Glucose Mannitol Salt media (GMS) [72], respectively.

For total RNA isolation using TriReagent (Sigma), 2 l flasks with 500 ml TY or GMS medium, supplemented with 8 μg/ml nalidixic acid, were inoculated with 2 ml of pre-culture and incubated in a rotary shaker (300 rpm) at 30°C to at OD_600 _= 0.6.

For RNA isolation with the miRNeasy Kit, 100 ml flasks with 50 ml TY or GMS medium, supplemented with 8 μg/ml nalidixic acid, were inoculated with 200 μl of pre-culture and incubated in a rotary shaker (175 rpm) at 30°C to an OD_600 _= 0.6.

Exponential and stationary phase samples without stress exposure were obtained from cultures in GMS and TY media at an OD_600 _of 0.6 for the exponential phase and OD_600 _of 2.8 and 3.5 for stationary phase samples.

For stress induction, the medium and growth conditions were modified as follows. High salt stress: addition of NaCl to a final concentration of 0.4 M in GMS medium. Oxidative stress: addition of H_2_O_2 _to a final concentration of 10 mM in GMS medium. Cold shock stress: temperature shift of the culture from 30°C to 20°C in TY medium. Heat shock stress: temperature shift of the culture from 30°C to 40°C in TY medium. Acid or alkaline stress: cultures grown in GMS to an OD_600 _of 0.6 were centrifuged and then re-suspended in GMS modified by adding HCl to pH 5.8, or by adding NaOH to pH 8.5. In each case, cells were harvested 15 and 45 min after exposure to stress conditions.

Total RNA used in Northern blot analysis was isolated from cultures grown in 100 ml flasks with 50 ml TY or GMS medium supplemented with streptomycin (250 μg μl^-1^) inoculated with 500 μl pre-culture and incubated in a rotary shaker (140 rpm) at 32°C to an OD_600 _of 0.6. These cultures were subjected to the following stress conditions. The TY cultures were stressed for 45 min at 40°C or 20°C; for salt-stress, 0.4 M NaCl was added to the GMX culture for 45 min; control cultures were incubated for the same time without stress.

### RNA Isolation procedures

#### Isolation of total RNA for 5'/3'-RACE (Rapid Amplification of cDNA Ends)

The cell pellet obtained from 50 ml culture were re-suspended in 6 ml TriReagent (Sigma). Cell disruption and homogenization was performed using the FastPrep-24 sample preparation system (MP). Incubation of the samples for 15 min at room temperature and centrifugation at 16,000 g for 15 min at 4°C were performed. Chloroform (0.2 vol) was added to the liquid upper phase and shaken vigorously for 30 sec followed by incubation for 3 min and centrifugation for 15 min at 16,000 g; 4°C. For precipitation 0.5 volume of high salt-precipitation solution (0.8 M Na-citrate/1.2 M NaCl) and 0.5 volume of isopropanol were added to the aqueous supernatant and shaken vigorously. Sample was further incubated at 20°C for 10 min and centrifuged for 10 min at 16,000 g; 4°C. The RNA pellet was washed two times with 75% (v/v) ethanol, air-dried and resuspended in 50 μl deionized water.

#### Isolation and fractionation of RNA for synthesis of cDNA used in deep sequencing and microarray hybridizations

Total RNA, including the small sized RNA, was isolated using the miRNeasy Mini Kit (Qiagen) according to the manufacturer's instructions. Subsequently, total RNA was purified with 2.5 volumes phenol:chloroform:isopropanol (PCI). RNA was isolated and separated into small RNA (< 200 nt) and long RNA (> 200 nt) fractions using the miRNeasy Mini Kit (Qiagen) or the mirVana miRNA Isolation Kit (Ambion) according to the manufacturers' instructions. Quality of all RNA samples was analyzed using the Agilent RNA 6000 Nano Kit on the Agilent 2100 Bioanalyzer (Agilent Technologies).

### Deep sequencing of cDNAs derived from sRNA transcripts

#### RNA treatment for primary and processed transcript sequencing

For enzyme treatment 80 μg of PCI purified total RNA was separated into 20 μg samples. Each sample was treated with 9 U of Terminator Phosphate Dependent Exonuclease (TPE) in 30 μl volumes for 2.5 h at 30°C. Following the treatment the samples were PCI purified. Pooled TPE treated samples were separated into 5 μg aliquots and further treated with tobacco acid pyrophosphatase (TAP) (2.5 U/5 μg RNA in 50 μl for 2 h). Finally, purification of the sample was carried out via PCI purification. Treated sample for primary transcript sequencing and untreated sample for processed transcript sequencing were analysed with the Agilent Bioanalyzer system as described above.

#### Total RNA treatment for transcriptome analysis

Total RNA sample was treated with TAP and purified in the same way as described above.

#### cDNA-Library preparation and sequencing applying the Genome Analyzer II (Illumina/Solexa)

TAP treated sample of total RNA was further processed by GATC Biotech (Konstanz, Germany) in the following steps: (i) 5'-and 3'-RNA adapters were ligated to the RNA for first-strand cDNA synthesis and second strand amplification via PCR. After double-strand DNA synthesis, RNA and free adapters were removed and the remaining cDNA was purified. (ii) Nebulization of DNA, ligation of sequencing specific adapters and separation of 150 to 200 nt sized DNA fragments were carried out. (iii) Sequencing with a read length of 36 nt was carried out by GATC Biotech using the Genome Analyzer II (Illumina/Solexa). Data analysis and base calling were performed applying the Illumina instrument software.

#### DNA-Library preparation and sequencing applying the Genome Sequencer FLX (Roche)

TAP and TPE treated sample and untreated sample were further processed by GATC Biotech in the following steps: (i) polyacrylamide gel electrophoretic separation, excision and purification of RNA within a range of 50 to 350 nt, (ii) poly-A tailing and adapter ligation for first-strand cDNA synthesis and second strand PCR amplification, (iii) following the pooling of both samples each set was differentially tagged with dsDNA adapters, (iv) immobilization on beads, emulsion PCR, and sequencing to a maximum of 400 nt in length was carried out by GATC Biotech applying the Titanium Kit on the GS FLX Sequencing System (Roche).

### RNA and cDNA labeling for microarray and Affymetrix Symbiosis Chip hybridizations

#### Labeling for microarray hybridizations

Cy3- and Cy5-labeled cDNA or RNA fragments directed against transcripts derived from the plus and the minus genomic DNA strand were generated from the same fractions of small RNA and long RNA pools. RNA was directly labeled by PolyA polymerase-dependent 3'-tailing using the mirVana miRNA Labeling Kit (Ambion). After tailing, cDNA synthesis was carried out as previously described using oligo-dT and aminoallyl random hexamer primers [73].

#### cDNA-labeling for Affymetrix Symbiosis Chip

cDNA-synthesis derived from small RNA fractions (< 200 nt) was performed after polyA-tailing of the small RNAs by reverse transcription using biotin-modified random hexamers and oligo-dT primers, and dNTPs including biotin-dUTP. cDNA extracts from both exposure times of each stress condition were pooled. Additionally, the exponential and stationary phase RNA preparations each from GMX and TY media were pooled.

### Hybridization and image acquisition of microarrays and Affymetrix Symbiosis Chips

#### Microarray hybridization and image acquisition

Hybridization of the small RNA fraction (Cy3-fluorescent marker) was compared to that of the long RNA fraction (Cy5-fluorescent marker). Three combinations were performed: (i) the small RNA fraction with the long RNA fraction, both of which were directly labeled by 3'-tailing, (ii) labeled cDNAs obtained from both RNA fractions, and (iii) a combination of directly labeled small RNA fraction and labeled cDNA derived from the long RNA fraction. Two or three biological and at least two technical replicates were made for each condition and time point. Microarray processing, sample hybridization, and image acquisition were performed as described previously [73] applying the Sm14kOLI microarray that carries 50 mer to 70 mer oligonucleotide probes directed against coding regions and both strands of the intergenic regions (*Sinorhizobium meliloti *1021 Sm14kOLI) [32]. Probes in intergenic regions were separated by approximately 50 to 100 nt. Analysis of microarray images was performed with ImaGene 6.0 software (BioDiscoveries) [73]. Lowess normalization and significance test (fdr) were performed with the EMMA software [74]http://www.cebitec.uni-bielefeld.de/groups/brf/software/emma_info/. The M-value represents the logarithmic ratio between both channels. The A-value represents the dual logarithm of the combined intensities of both channels. Oligonucleotide probes with positive M-values ≥ 3 indicate an 8-fold enrichment of small RNA fragments (≤ 200 nt) and therefore were classified as markers for sRNA candidates.

#### Affymetrix GeneChip hybridization and image acquisition

Sample hybridization was applied with 4 μg of biotinylated cDNA and the dual genome SymbiosisChip [31,75] according to the Affymetrix GeneChip Expression analysis technical manual [Affymetrix GeneChip Expression analysis technical manual, chapter 6 Prokaryotic Target Hybridization] but with the following modifications: (i) staining and washing steps were performed with respect to the standard fluidics program FlexFS450 on Fluidics Station 450 (Affymetrix) except that the washing step with buffer B was at 48°C (instead of 45°C). This chip contained 25 mer probes that were evenly separated by less than eight nucleotides covering both strands of intergenic regions larger than 150 nt and probe sets for the predicted coding regions of *S. meliloti*, and in addition 9,935 probe sets representing *Medicago truncatula *ESTs [31,75].

Images were acquired using the GeneChip Scanner3000 7G (Affymetrix). Analyses of the images were performed with the GCOS 1.4 software (Affymetrix). Normalization was calculated using the dChip 2008 software (Affymetrix). Two parameters were determined to define signals marking putative small non-coding RNAs: (i) a signal intensity at least two-fold higher than the signal intensity of the background; (ii) at least two significant signals originating from two different probes located in the same intergenic region at a maximum distance of 200 nt.

### 5'-RACE

5'-RACE was performed as described by Argaman et al. (2001) with the following modifications: (i) 12 μg total RNA was treated with 10 to 25 units TAP at 37°C for 120 min to eliminate pyrophosphates from primary transcript 5'-triphosphates; (ii) 1 nmol 5'-adapter (5'GUA UGC GCG AAU UCC UGU AGA ACG AAC ACU AGA AGA AA3', Operon) was ligated using T4 RNA ligase (Fermentas) at 37°C for 4 h in a buffer containing 50 mM HEPES-NaOH (pH 8.0 at 25°C), 10 mM MgCl_2_, 10 mM Dithiothreitol (DTT), 1 mM ATP and 0.05 mg/ml bovine serum albumin (BSA) (reaction was stopped with PCI extraction and ethanol precipitation); (iii) the remaining RNA (5-8 μg) was reverse transcribed with the Superscript III reverse transcriptase (Invitrogen) using a gene specific primer according to the manufacturer's instructions but for 120 min incubation time at 55°C, instead of 60 min; (iv) products were amplified using the HotStar Taq Mastermix Kit (Qiagen) or Taq-DNA polymerase (Thermo Fisher) according to the manufacturer's instructions using gene- and adapter-specific primers [Additional file 12] applying the following PCR program: 95°C/15 min; 40 cycles of 95°C/40 sec, 65°C/40 sec, 72°C/40 sec and finally, 72°C/10 min; (v) cloning of the resulting fragments into plasmid vector pCR 2.1-TOPO (Invitrogen). Cloned 5'-RACE-products were sequenced by GATC Biotech using the ABI 3730 XL Sequencing System.

### Northern hybridizations

The cells were harvested at 0°C for 10 min at 6,000 g. Pellets were frozen in liquid nitrogen and stored at -80°C. RNA isolation, separation, Northern blotting and the probe for detection of 5S rRNA were previously described [76]. Briefly, cells were disrupted with glass beads (Sigma) and Ribolyser (Hybaid), and total RNA was isolated with TRIzol (Invitrogen), followed by additional extraction with water-saturated hot phenol (65°C), phenol-chloroform and chloroform-isoamylalcohol. The precipitated RNA was dissolved in water.

RNA samples (15 μg) were denatured in urea-formamide loading buffer [77] for 15 min at 65°C, placed on ice and loaded on 1 mm thick 10% polyacrylamide-urea gels [77]. Separation was performed for 2 h at 400 V. RNA was then transferred onto a nylon membrane (Pall) for 2 h at 400 mA or overnight at 50 mA using a semidry blotter (Peqlab), and hybridized with oligonucleotides, which were radioactively labeled at the 5'-end using gamma^32^P-ATP and polynucleotide kinase. After overnight hybridization at 56°C in solution containing 10 pmol labeled primer (approx. 2 to 5 × 10^6 ^c.p.m.), 6 × SSC, 0.5% SDS and salmon sperm DNA, the membranes were washed twice in 0.01% SDS, 5 × SSC at room temperature. Membranes were stripped for 20 min at 96°C in 0.1% SDS and re-hybridised up to four times. DNA-oligonucleotides used for hybridization are listed in [Additional file 12].

Signals were detected and analysed using a BioRad molecular imager and the Quantity One (BioRad) software. The intensity of the sRNA bands was normalized to the intensity of the 5S rRNA. RNA probes from two independent TY controls were on each blot - a control for the heat shock and a control for the cold shock experiment.

### Bioinformatics data analysis

#### Read mapping and transcript classification

Read mapping to the *S. meliloti *genome was performed with the SEGEMEHL software [78] using the following parameters: extension penalty = 2, differences = 1, accuracy = 90. Criteria of assembling reads into transcripts were defined as described in the main text, using parameters (L, C, c) = (50-350,10,5) for 454 and (L, C, c) = (50,5,2) for Illumina/Solexa data. Initial read mapping and visualization was carried out applying the MapView Program (Sebastian Jänicke, Bielefeld University, Germany).

Distinct 5'- and 3'-ends were determined as follows: (i) a distinct 5'-end within the first two bases of a contig requires more than 5 reads sharing the same end, (ii) subsequent distinct 5'-ends additionally require a number of reads sharing the same 5'-end which represents at least 10% of the mean number of reads covering the contig, (iii) symmetric rules apply for distinct 3'-ends.

#### Promoter prediction

A position specific scoring matrix (PSSM) was derived from 25 experimentally verified promoter sequences provided in MacLellan *et al*. [41] with pseudocount 0.01. Promoter search was performed with the PoSSuMsearch program [79] at P-value cutoff 2.155343e-11.

#### Confirmation of known (annotated) sRNAs among transcripts

This was achieved by identifying transcripts, which were mapped to genomic loci annotated as sRNA.

#### BLAST Homology Search

BLAST searches were performed using NCBI BLAST 2.2.19 [80] and the GenBank, EMBL, DDBJ, and PDB databases as of Oct.28, 2009, using a relaxed E-value cutoff of E = 0.02. Results for more stringent cutoffs were obtained by filtering on the E-value of the hits.

#### Small protein analysis

After identifying potential open reading frames, we applied the free_align software [81] with energy threshold -3.4535 and UCCUCCA for the 16S RNA tail in *S. meliloti *to predict ribosomal binding sites.

#### Structural analysis

RNA transcripts were folded with *RNAshapes*, using complete probabilistic folding mode, the most abstract shape level 5, and default parameters. An abstract shape is e.g. a single hairpin [], a cloverleaf [[][][]] or a Y-shape [[][]]. The shape probability is the accumulated Boltzmann probability of all (optimal or suboptimal) foldings of the RNA which exhibit this shape. (Shape probabilities are preferable to folding energies as they are independent of sequence size and base composition.) Each shape holds a secondary structure of minimum free energy as its representative.

#### Shape probability Z-scores

To evaluate whether our transcripts have more well-defined structures than randomly picked sequences of the same length from the *S. meliloti *genome, we computed Z-scores as follows: Let transcript t have length n and the most likely (or dominant) shape p with Prob(t, p) = x. For all sequence windows of length n on the same strand as t in the *S. meliloti *genome, we compute those windows w which also have shape p as their dominant shape. This gives a distribution of values Prob(w, p) with expectation E(p, n) and standard deviation S(p, n). The Z-score of transcript t is defined (as usual) by (Prob(t, p) - E(p, n))/S(p, n). Comparing this approach to the literature, it is important to note that it is different from approaches which consider folding energy. In that context, a Z-score of -4 or lower is necessary to indicate a significant folding energy [82]. Here, however, we compare to a background of *dominant *shapes.

#### Rfam homology search

Rfam searches were performed with the gathering cut-offs suggested by Rfam, version 9.1, and the Rfam HMM filtering turned on. To reduce the computation time for the Rfam search with 1,080 transcripts derived from 454 sequencing, the shape filtering method implemented by *RNAsifter *[47] was employed with default parameters.

#### Refined Rfam homology search

For the refined Rfam search, we modified the *RNAsifter *pipeline in the following way: *RNAsifter *uses an abstract shape index of Rfam and first computes the four most likely abstract shapes for each query, on different levels of shape abstraction. In a case where the query has a perfect shape match to Rfam family X, we call the Rfam covariance model for X with the HMM filter turned off. We record a match if the score is larger than 0.25 * T, where T is the recommended gathering threshold.

## Abbreviations

BSA: bovine serum albumin; C: coverage; c: extended coverage; cDNA: copyDNA; COG: clusters of orthologous groups; DTT: dithiothreitol; EST: expressed sequence tags; GMS: glucose mannitol salt media; HMM: hidden markov model; IGR: intergenic region; IS element: insertion element; L: length; nt: nucleotide; OD: optical density; ORF: open reading frame; PCI: phenol chloroform isoamylalcohol; PCR: polymerase chain reaction; REP element: repetitive extragenic palindromic element; RBS: ribosomal binding site; RACE: rapid amplification of cDNA ends; ROSE: repression of heat-shock gene expression; rpm: rounds per minute; sRNA: small non-coding RNA; TAP: tobacco acid pyrophosphatase; TPE: terminator phosphate dependent exonuclease; TY: tryptone yeast; UTR: untranslated region; vol: volume

## Authors' contributions

JPS and JR contributed equally to this investigation. JPS and JR carried out the predominant experimental and data analysis part, respectively, of the study and drafted the manuscript. StJ developed the refined Rfam search. EEH and JB performed Northern blot and Affymetrix Symbiosis Chip hybridizations, respectively, and SD carried out a part of the microarray experiments. SeJ helped with an initial read mapping for the 454 data, developed and provided the MapView program. AB designed and generated the microarrays. AB and RG designed and coordinated the study and drafted the manuscript. All authors read and approved the final manuscript.

## Supplementary Material

Additional file 1 **Table S1: sRNA candidates derived from deep sequencing**. This table lists the sRNAs identified by 454 and SolexA/Illumina cDNA sequencing in this study. It includes position, size, classification, sequence coverage, and identified end data as well as the associated genes of sense and antisense sRNAs. Click here for file

Additional file 2 Table S2: Promoter predictions. Click here for file

Additional file 3 **Table S3: Homologous regions related to REP and transposable elements with transcriptional activity**. This table lists REP elements with transcriptional activity and sRNA candidates located in transposable elements. It includes position, size, classification, and the associated repetitive or transposable element. Click here for file

Additional file 4 **Table S4: Microarray data**. This table summarizes the microarray data of sRNAs identified by this approach. Click here for file

Additional file 5 Table S5: Affymetrix Symbiosis Chip hybridizations data of putative sRNA regions identified by this approach. Click here for file

Additional file 6 **Table S6: **Sorting of microarray data of sRNA candidates identified in at least one of the conditions analyzed by microarray hybridizations. Click here for file

Additional file 7 **Table S7: Small protein analysis**. sRNA candidates with open reading frames larger than 60 nt. Click here for file

Additional file 8 **Table S8: Predicted sRNA structures of short derivatives of full length sRNAs shown in Figures**11** and**12 Click here for file

Additional file 9 **Table S9: BLAST search results**. BLAST results for different cut-off criteria as shown in Table 3 are given. Click here for file

Additional file 10 Table S10: Secondary structure predictions of trans-encoded sRNAs. Click here for file

Additional file 11 Results of Rfam homology and Rfam refined homology search. Click here for file

Additional file 12 Table S12: Primer and probe sequences for 5'-RACE and Northern Blot experiments. Click here for file
